# An allosteric inhibitor of sirtuin 2 deacetylase activity exhibits broad-spectrum antiviral activity

**DOI:** 10.1172/JCI158978

**Published:** 2023-06-15

**Authors:** Kathryn L. Roche, Stacy Remiszewski, Matthew J. Todd, John L. Kulp, Liudi Tang, Alison V. Welsh, Ashley P. Barry, Chandrav De, William W. Reiley, Angela Wahl, J. Victor Garcia, Micah A. Luftig, Thomas Shenk, James R. Tonra, Eain A. Murphy, Lillian W. Chiang

**Affiliations:** 1 Evrys Bio LLC, Pennsylvania Biotechnology Center, Doylestown, Pennsylvania, USA.; 2 Department of Molecular Genetics and Microbiology, Duke Center for Virology, Duke University School of Medicine, Durham, North Carolina, USA.; 3 International Center for the Advancement of Translational Science, Division of Infectious Diseases, Center for AIDS Research, University of North Carolina, School of Medicine, Chapel Hill, North Carolina, USA.; 4 TICRO Bioservices, Trudeau Institute, Saranac Lake, New York, USA.; 5 Department of Molecular Biology, Princeton University, Princeton, New Jersey, USA.; 6 Microbiology and Immunology Department, SUNY Upstate Medical University, Syracuse, New York, USA.

**Keywords:** Infectious disease, Virology, Drug therapy, Epigenetics, Structural biology

## Abstract

Most drugs used to treat viral disease target a virus-coded product. They inhibit a single virus or virus family, and the pathogen can readily evolve resistance. Host-targeted antivirals can overcome these limitations. The broad-spectrum activity achieved by host targeting can be especially useful in combating emerging viruses and for treatment of diseases caused by multiple viral pathogens, such as opportunistic agents in immunosuppressed patients. We have developed a family of compounds that modulate sirtuin 2, an NAD^+^-dependent deacylase, and now report the properties of a member of that family, FLS-359. Biochemical and x-ray structural studies show that the drug binds to sirtuin 2 and allosterically inhibits its deacetylase activity. FLS-359 inhibits the growth of RNA and DNA viruses, including members of the coronavirus, orthomyxovirus, flavivirus, hepadnavirus, and herpesvirus families. FLS-359 acts at multiple levels to antagonize cytomegalovirus replication in fibroblasts, causing modest reductions in viral RNAs and DNA, together with a much greater reduction in infectious progeny, and it exhibits antiviral activity in humanized mouse models of infection. Our results highlight the potential of sirtuin 2 inhibitors as broad-spectrum antivirals and set the stage for further understanding of how host epigenetic mechanisms impact the growth and spread of viral pathogens.

## Graphical abstract

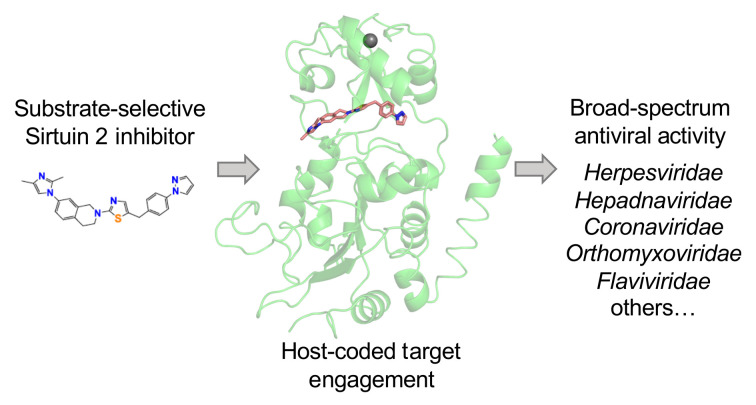

## Introduction

Direct-acting antivirals (DAAs) target virus-coded products and are a highly successful therapeutic paradigm. The sofosbuvir/velpatasvir combination, which inhibits the hepatitis C virus polymerase complex, is a case in point. It cures a high proportion of patients with chronic hepatitis C (1). However, DAAs suffer from 2 principal limitations. First, viruses develop resistance, as is well documented for influenza M2 ion channel and neuraminidase inhibitors (2). Second, although there are exceptions (3), DAAs generally target only one virus or virus family.

In contrast to DAAs, host-targeted antivirals (HTAs) inhibit a host product that supports viral replication or enhance the activity of a defensive network. With no selective pressure for the accumulation of mutations in host cell antiviral targets, viral resistance should not develop. Further, since different viruses use overlapping cellular pathways and factors to support their replication (4) and antiviral defense systems often target these common pathways, HTAs can exhibit broad-spectrum activity (5). Thus, HTAs have potential to treat categories of viral disease where the causative agents span multiple virus families. Importantly, broad-spectrum HTAs have potential to provide a rapid therapeutic solution at the onset of a pandemic, reducing the time between novel virus identification and pharmacological intervention (6, 7). Beyond this periodic need, HTAs can treat patients at risk for infection with viruses of different families, such as transplant patients who are at elevated risk for herpesvirus, paramyxovirus, polyomavirus, hepadnavirus, and coronavirus infections during immunosuppressive therapy (8, 9).

One intriguing network of targets for the development of HTAs are the proteins that create and read the cellular acetylome. Lysine N-ε-acetylations are found on thousands of proteins (10). Histone acetyltransferases (HATs) transfer an acetyl group from acetyl-coenzyme A to a target lysine, bromodomain-containing proteins read these modified lysine residues to regulate diverse cellular processes, and histone deacetylases (HDACs) remove the marks. The dynamic interplay between HATs and HDACs specifies the acetylome, which impacts chromatin structure and transcriptional activity, protein-protein interactions, protein localization, and metabolic processes (11, 12).

Not surprisingly, given their broad impact on cellular processes, lysine N-ε-acetylations modulate factors critical for viral replication. For example, transcription of the HIV genome is influenced by histone acetylation, the viral integrase and transactivator of transcription (Tat) proteins are regulated by acetylation, and HIV latency is modulated by drugs that inhibit deacetylation (13). Likewise, influenza A proteins are regulated by acetylation (14), and human cytomegalovirus (HCMV) infection profoundly impacts the cellular and viral acetylomes (15). In addition to supporting viral processes, lysine acetylation also impacts cellular antiviral defense systems. For example, the location of the DNA sensor protein IFN-γ–inducible protein 16 (IFI16) is controlled by acetylation (16), and the activity of nuclear factor-κB (NF-κB), which regulates numerous elements of immune responses, is modulated via acetylations within its p65 subunit (17).

The inhibition of deacetylation has been explored as an antiviral mechanism. HDACs form 2 major families: Zn^++^-dependent HDACs and NAD^+^-dependent HDACs, also termed sirtuins (SIRTs). The seven SIRTs (SIRT1–SIRT7) (18) transfer an acyl group from an acylated lysine of a protein substrate to the ADP-ribose moiety of NAD^+^, deacylating the protein and producing nicotinamide plus 2′-*O*-acyl-ADP-ribose (19). Deacetylation is most commonly studied, but SIRTs also remove longer-chain acyl groups (20). The NAD^+^ requirement ties SIRT activity to the metabolic state of cells, and infection significantly disrupts cellular metabolism (21, 22). The human SIRTs are localized to distinct cellular compartments (23), and knockdown of each human SIRT, as well as the *E.* *coli* SIRT CobB, modulates the growth of multiple viruses (24), underscoring their evolutionarily conserved roles in the control of viral replication. The dual SIRT1/2 inhibitors tenovin-1 and sirtinol inhibit the growth of both RNA and DNA viruses (25, 26); and the SIRT2-selective inhibitor AGK2 (27) antagonizes the replication of hepatitis B virus (28, 29). SIRT2-knockout mice are healthy and immunocompetent (30, 31), arguing that selective SIRT2 inhibitors are likely to be well tolerated as antiviral therapeutics.

Here we explore the utility of small molecules targeting SIRT2 as broad-spectrum antivirals with potential to treat opportunistic infections. We demonstrate that the compound FLS-359 binds to SIRT2 and allosterically inhibits its deacetylase activity. Broad-spectrum antiviral activity of the drug is evident across multiple DNA and RNA virus families, including the herpesviruses, human cytomegalovirus (HCMV), and Epstein-Barr virus (EBV), which are of particular concern in immunosuppressed patients (32–36). The drug reduces the accumulation of HCMV RNAs and DNA, substantially decreasing virus spread and infectious yield in human fibroblasts. FLS-359 also reduced virus production in 2 humanized mouse models of HCMV infection. These results highlight SIRT2 as a host target, and support further development of drugs targeting SIRT2 as broad-spectrum antivirals.

## Results

### FLS-359 binds SIRT2, selectively reducing deacetylase activity.

**Figure 1:**
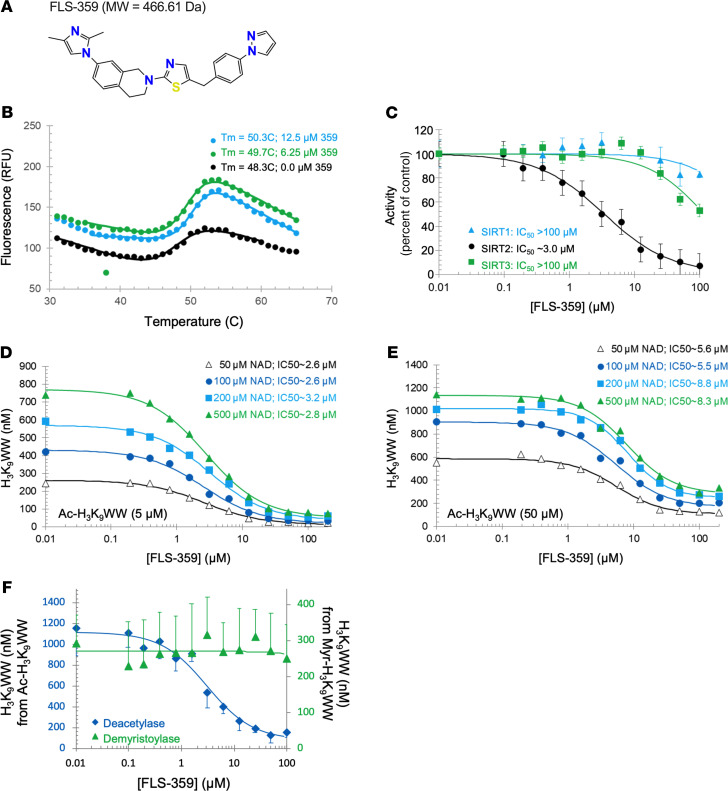
FLS-359 binds SIRT2, selectively reducing its catalytic activity. (**A**) Structure of FLS-359, 7-(2,4-dimethyl-1*H*-imidazol-1-yl)-2-(5-{[4-(1*H*-pyrazol-1-yl)phenyl]methyl}-1,3-thiazol-2-yl)-1,2,3,4-tetrahydroisoquinoline. (**B**) FLS-359 alters SIRT2 thermal stability. Recombinant human SIRT2^2–389^ (2.5 μM) denaturation was assayed in the presence of solvent (black), 6.25 μM (green), or 12.5 μM FLS-359 (blue). (**C**) FLS-359 exhibits SIRT2-selective activity. Reactions used recombinant human SIRT1^1–747^ (blue), SIRT2^2–389^ (black), or SIRT3^1–400^ (green) protein (*n* = 3). (**D** and **E**) SIRT2 inhibition by FLS-359 is not highly dependent on substrate concentration. NAD^+^ concentration was varied from *K_m_* (50 μM) to 10× *K_m_* (500 μM). Deacetylase assays used Ac-H_3_K_9_WW at *K_m_* (5 μM, **D**) or 10× *K_m_* (50 μM, **E**). (**F**) FLS-359 does not inhibit SIRT2 demyristoylase activity. Reactions used 5 μM acylated peptide (Myr-H_3_K_9_WW) and 500 μM NAD (*n* = 3).

Starting with a hit from a small-molecule screen designed to identify compounds that altered SIRT2 activity in vitro, we developed a portfolio of molecules targeting SIRT2 using anti-HCMV activity as a primary criterion in our lead optimization campaign. FLS-359, 7-(2,4-dimethyl-1*H*-imidazol-1-yl)-2-(5-{[4-(1*H*-pyrazol-1-yl)phenyl]methyl}-1,3-thiazol-2-yl)-1,2,3,4-tetrahydroisoquinoline (Figure 1A), is a representative lead.

An in vitro thermal shift assay (37) was used to test for FLS-359–SIRT2 binding by quantifying the compound-dependent increase in protein thermal stability (Figure 1B). SIRT2 underwent thermal denaturation with a midpoint transition temperature (*T_m_*) of approximately 48°C. FLS-359 increased the *T_m_* by 1.4°C (6.25 μM) or 2.0°C (12.5 μM), demonstrating that the drug engages and stabilizes the purified protein.

The effect of FLS-359 on deacetylation of a peptide containing a histone H3 acetylation site (Ac-H_3_K_9_WW) was assayed in vitro, using mass spectrometry to quantify deacetylated peptide. The concentration for half-maximal inhibition (IC_50_) in this assay was about 3 μM for SIRT2 and more than 100 μM for SIRT1 and SIRT3 (Figure 1C). When the NAD^+^ concentration was increased from 50 to 100, 200, and 500 μM, more deacetylated product was generated, but the IC_50_ remained essentially the same (Figure 1, D and E). The IC_50_ was about 3 μM for all NAD^+^ concentrations when the peptide was used at 5 μM. The IC_50_ was about 7 μM for all NAD^+^ concentrations at 50 μM peptide. Therefore, FLS-359 is not competitive with NAD^+^. When the peptide concentration was increased from 5 μM (1× *K_m_*; Figure 1D) to 50 μM peptide (10× *K_m_*; Figure 1E), the IC_50_ increased from about 3 μM to about 7 μM, or about 2-fold. If FLS-359 binding was fully competitive with the peptide, an approximately 10-fold increase in IC_50_ would be expected. In addition, saturating compound decreased SIRT2 activity to a lower but residual value. Both of these observations are consistent with partial inhibition, seen with other SIRT2 inhibitors, e.g., AGK2 (27) and MIND4 (38). Since SIRT2 is important to cellular metabolic homeostasis, the observed partial inhibition may prove to be a positive attribute that supports cell viability in uninfected cells while providing an antiviral effect in infected cells.

SIRT2 has deacylation activities in addition to deacetylation (20, 39), including demyristoylation, and several SIRT2 inhibitors are acyl group selective, blocking deacetylation but not demyristoylation (40–43). When tested for inhibition of demyristoylation using the same peptide backbone (Myr-H_3_K_9_WW) used to assay deacetylation (Ac-H_3_K_9_WW), neither FLS-359 nor tool compounds (AGK2 and SirReal2) showed activity (Figure 1F and Supplemental Table 1; supplemental material available online with this article; https://doi.org/10.1172/JCI158978DS1). Thus, FLS-359 is substrate selective, inhibiting deacetylation but not demyristoylation.

### FLS-359 induces known activities of SIRT2 modulators within cultured cells.

SIRT2 deacetylates α-tubulin K40 (44); and, as expected, treatment of cultured HepG2 hepatocellular carcinoma cells with FLS-359 for 24 hours increased the level of acetylated α-tubulin in HepG2 cells by a factor of about 3 (Supplemental Figure 1A). SIRT2 knockdown or inhibition induces degradation of c-Myc protein in tumor cells by inducing its ubiquitination (45, 46), and treatment with FLS-359 for 72 hours dramatically reduced the level of the oncoprotein in MDA-MB-231 breast adenocarcinoma cells (Supplemental Figure 1B). Notably, c-Myc levels were not changed by treatment of MRC-5 human diploid fibroblasts with the drug (Supplemental Figure 1C). Thus, treatment with FLS-359 induces known consequences of SIRT2 inhibition in tumor cells.

### X-ray structure confirms FLS-359 engagement of SIRT2.

X-ray crystallographic structures have been determined for human SIRT1, 2, 3, 5, and 6 (47). The structures consist of 2 domains: an upper Zn^++^-binding domain and a lower catalytic domain with a Rossmann fold (48, 49). The 2 domains behave as a clamshell, accommodating varying acyl-Lys modifications within a flexible pocket between the 2 clamshell domains. This area is above the C pocket of the NAD^+^-binding site and is termed the extended C (EC) pocket (49). SIRT1, SIRT2, and SIRT3 are about 70%–80% conserved around the NAD^+^ and nearby EC pockets, where natural and synthetic small-molecule ligands that affect activity have been resolved (47, 49).

**Figure 2:**
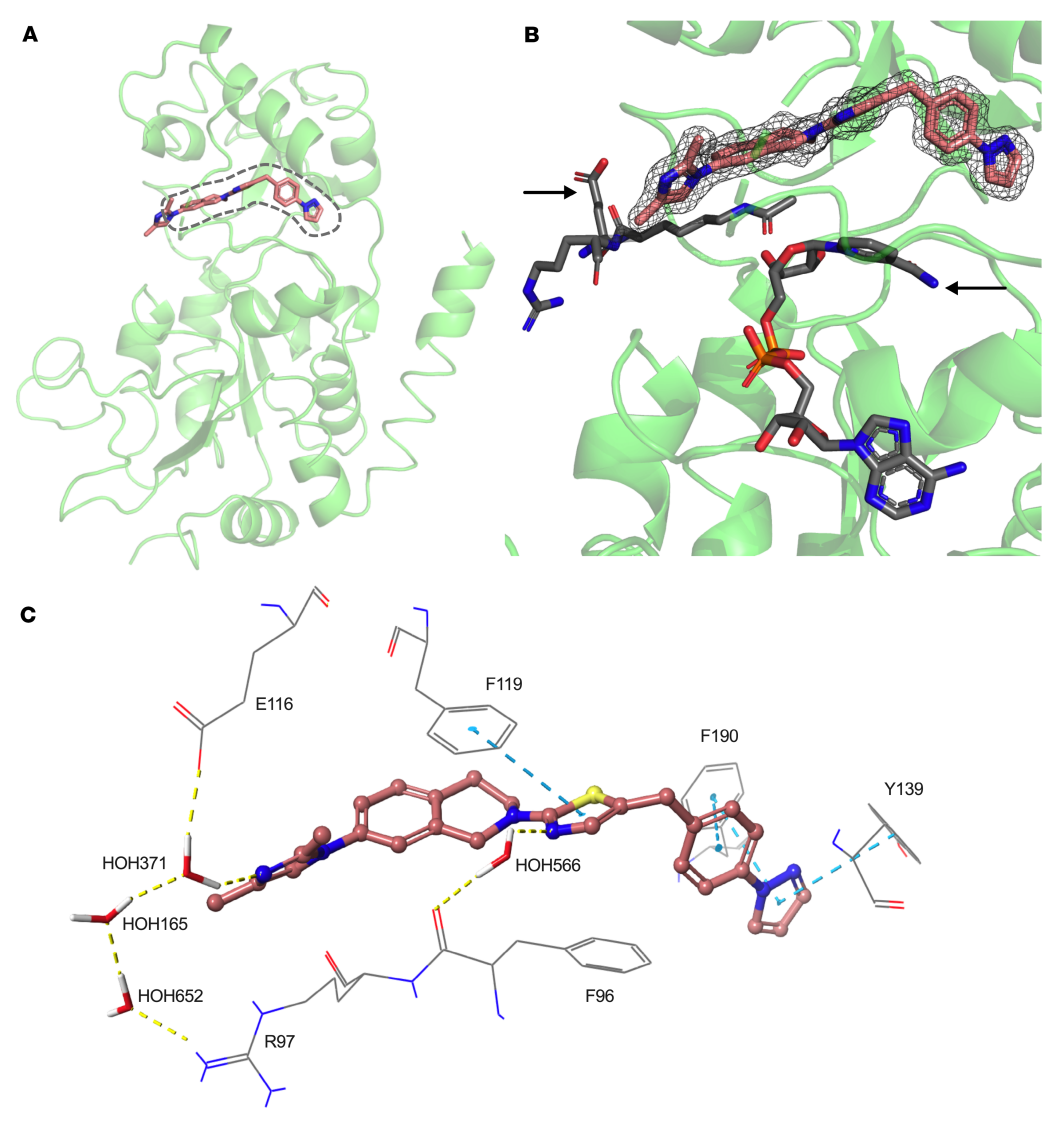
Co-crystal structure of FLS-359 bound to human SIRT2. (**A**) SIRT2 (green ribbon) with FLS-359 (pink carbon atoms in stick display) bound to its EC site (dashed lines). (**B**) Superposition of SIRT2-359 with an Ac-Lys peptide (right-pointing arrow; PDB ID 4RMI) plus NAD^+^ (left-pointing arrow; PDB ID 4RMG). The gray mesh over FLS-359 marks the 2*F_o_*–*F_c_* electron density map (contoured at 1.5 σ) resulting from refinement of the final model with REFMAC5. (**C**) Close-up view of the SIRT2 EC site focusing on the key FLS-359–SIRT2 interactions. Yellow-highlighted dashed lines indicate hydrogen bonds, and blue dashed bonds represent π-π interactions.

We produced crystals of FLS-359 bound to SIRT2 that diffracted to 1.8 Å resolution and determined the structure of the complex (Supplemental Table 2). FLS-359 sits in the SIRT2 EC pocket (Figure 2, A and B). In comparison with the unliganded SIRT2 apo structure (ref. 50; Protein Data Bank [PDB] ID 3ZGO), the clamshell has opened and an ordered α-helix has shifted to an unstructured loop (residues 294–304); in addition, a loop over the drug binding site is rearranged (Supplemental Figure 2). The 2 substrates, acetyl-Lys (PDB ID 4RMI) and NAD^+^ (PDB ID 4RMG), are computationally superimposed in Figure 2B. NAD^+^ binding is not predicted to be affected by FLS-359 binding. In contrast, the FLS-359 dimethylimidazole moiety resides in the same location as the peptidic acetyl group, but computational superpositioning predicts that the pocket can accommodate both FLS-359 and the peptide. The binding interactions of FLS-359 with SIRT2 include π-π interactions with residues F119, F190, and Y139 (Figure 2C). Water residue 566 bridges FLS-359 and SIRT2 with one hydrogen bond to the thiazole of FLS-359 and another hydrogen bond to the backbone carbonyl of residue F96. A multi-body water network in the peptide channel connects, via a hydrogen bond network, the dimethyl imidazole of FLS-359 to E116 through water residue 371 and to the side chain of R97 through water residues 371, 165, and 652. These water-mediated hydrogen bonds could be key interactions that drive binding affinity to SIRT2 in the hydrophobic EC site.

The FLS-359/SIRT2 structure was used to probe the substrate-selective activity of the drug. Flexible protein docking confirmed that acetyl-Lys (PDB ID 4RMI) and FLS-359 can simultaneously bind within the SIRT2 EC pocket (Supplemental Figure 3A), whereas peptides with thiomyristoyl lysine (PDB IDs 4R8M and 4X3P) are predicted to compete for binding with the drug (Supplemental Figure 3, B and C). Thus, myristoylated peptides have potential to exclude FLS-359 binding, providing an explanation for the drug’s substrate-selective activity.

In sum, enzyme thermal denaturation and kinetics studies (Figure 1), as well as co-crystal structure determination (Figure 2), argue that FLS-359 binds selectively to SIRT2 and induces an allosteric rearrangement of the active site, reducing the rate of deacetylation. It is a substrate-selective inhibitor, blocking deacetylation but not demyristoylation.

### FLS-359 exhibits broad-spectrum antiviral activity.

FLS-359 was tested for activity against multiple RNA and DNA viruses in cultured cells. The drug inhibited the growth of each pathogen shown (Table 1). The IC_50_ varied across the viruses, but the differences must be interpreted with caution, because the assays were performed at different research sites and used a variety of host cells. Nevertheless, in multiple cases, the antiviral IC_50_s were in the range of current standards of care, with acceptable half-maximal cytotoxic concentrations (CC_50_s). Most assays were performed in primary cells or diploid cell lines, because SIRT2 inhibition is antiproliferative or cytotoxic to many tumor cell lines (46, 51).

Given the importance of EBV and HCMV as adventitious agents in immunosuppressed patients (32–36), the effects of FLS-359 were examined in greater detail for these viruses. A broad-spectrum antiviral able to treat both infections has substantial potential utility.

### FLS-359 inhibits EBV lytic reactivation and/or replication.

**Figure 3:**
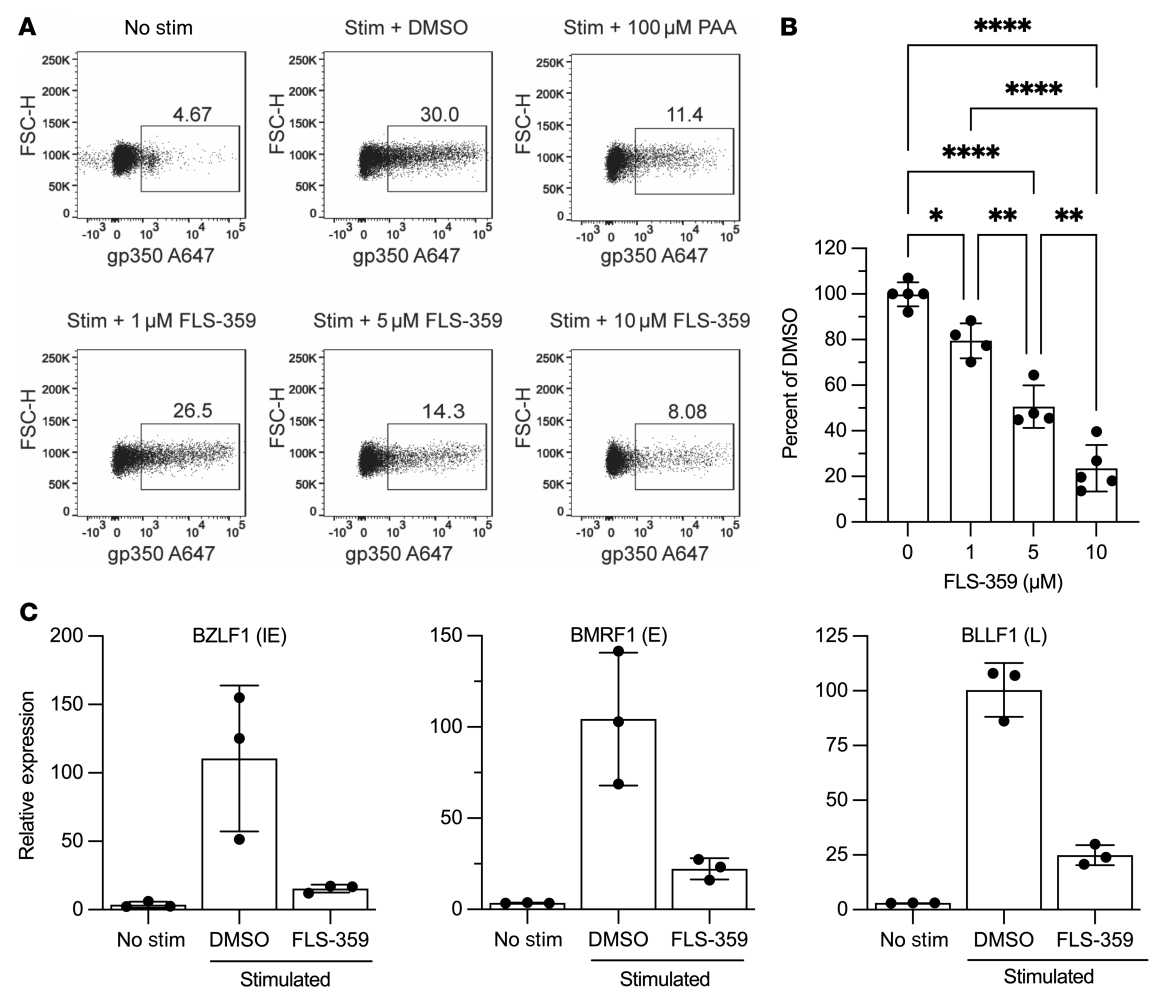
FLS-359 suppresses EBV lytic reactivation. (**A**) Akata BL cells were stimulated with 10 μg/mL anti-IgG and simultaneously treated with DMSO, 100 μM phosphonoacetic acid (PAA; positive control), or increasing concentrations of FLS-359 (1, 5, and 10 μM). Surface expression of the viral late gene gp350 was measured at 24 hours after induction by flow cytometry. (**B**) Dot plots reporting experiments in **A**, shown with mean ± SD (*n* = 4). *P < 0.05, **P < 0.01, and ****P < 0.0001. (**C**) Dot plots reporting quantitative reverse transcriptase PCR (qRT-PCR) measurement of BZLF1 (IE, immediate early), BMRF1 (E, early), and BLLF1 (L, late) gene expression suppressed by FLS-359 (10 μM) in Akata BL cells induced with anti-IgG. Mean ± SD is shown (*n* =3).

The Akata Burkitt lymphoma cell line is permissive for EBV lytic reactivation through activation of the B cell receptor (52). As expected, 24 hours after receptor engagement with anti-IgG, 30% of Akata cells induced surface expression of the late EBV gene gp350, which serves as a proxy for cells that are replicating viral DNA and forming new virions (Figure 3A). In the presence of the viral DNA replication inhibitor phosphonoacetic acid (PAA), gp350^+^ cells were reduced to about 11% (Figure 3A). FLS-359 treatment led to a dose-dependent decrease in gp350^+^ cells with 10 μM drug, inhibiting EBV reactivation at a similar level to PAA (Figure 3, A and B). The FLS-359 CC_50_ for Akata cells was greater than 100 μM (Table 1), so the inhibition of gp350 accumulation was not due to drug-induced cytotoxicity. The drug also inhibited the accumulation of viral immediate-early (BZLF1), early (BMRF1), and late (BLLF1) mRNAs (Figure 3C). We conclude that FLS-359 markedly reduces EBV lytic activation in Akata cells.

### FLS-359 inhibits HCMV spread in diploid fibroblasts.

**Figure 4:**
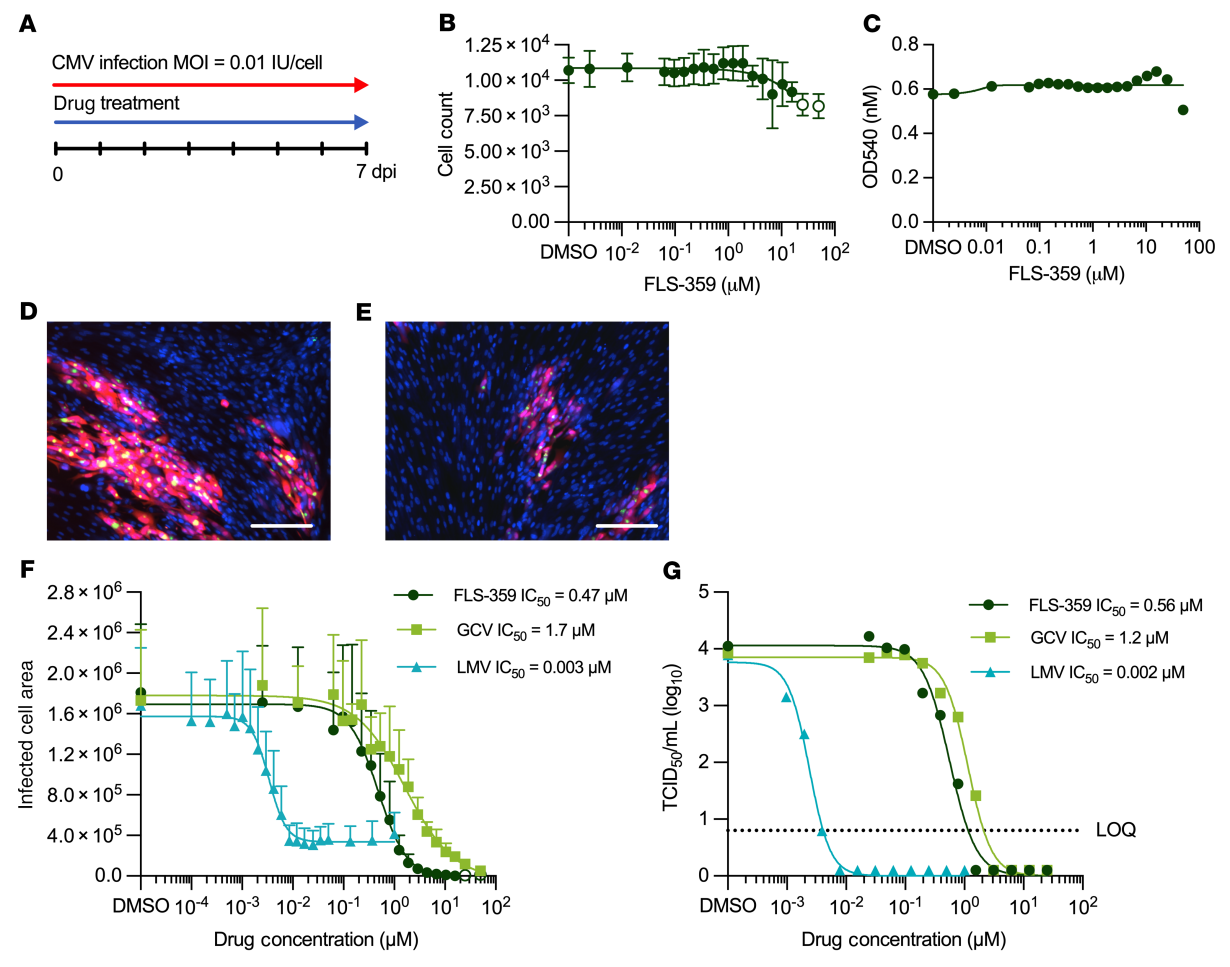
FLS-359 inhibits HCMV spread in fibroblasts. (**A**) Schematic representation of the spread assay. (**B** and **C**) Cytotoxicity was assessed in confluent MRC-5 cells after 7 days of FLS-359 treatment by DAPI staining for cell count (**B**) or neutral red uptake (**C**) (*n* = 3). (**D** and **E**) Confocal images of MRC-5 cells infected with TB40/E-mCherry-UL99eGFP (0.01 IU/cell) at 7 days post-infection (dpi), treated with vehicle (**D**) or FLS-359 at 0.5 μM (**E**). Fluorescent mCherry (red) is expressed with immediate-early kinetics and eGFP (green) with late kinetics, and DAPI (blue) locates nuclei. Scale bars: 300 μm. (**F**) Virus spread assay. CMV-infected cell area is quantified by mCherry fluorescence and plotted versus FLS-359, ganciclovir (GCV), or letermovir (LMV) concentrations. IC_50_ (mean ± SD) is reported (*n* = 4). (**G**) Virus yield assay. Cell-free virus at 7 dpi was quantified by TCID_50_. IC_50_ is reported. LOQ, limit of quantification.

The antiviral activity of a drug is generally measured by assaying of its effect on the production of infectious viral progeny. However, many viruses spread via 2 modes, either by release of a particle that eventually infects a new cell or by direct cell-to-cell transfer (53, 54). HCMV can move by either mechanism, and subviral particles can move from cell to cell, allowing the virus to spread without producing infectious particles (55). To capture the effect of drugs on HCMV movement and amplification by either mechanism, we used a spread assay. It uses a clinical isolate, TB40/E-mCherry-UL99eGFP (56), containing 2 reporters to monitor infection of MRC-5 fibroblasts: mCherry controlled by the SV40 early promoter, expressed with immediate-early kinetics, and eGFP fused to the viral UL99 coding region, expressed with late kinetics. The assay protocol is simple: infect confluent fibroblasts at a low input multiplicity (0.01 IU/cell), add drug immediately following infection, and quantify the area expressing fluorescent markers after 7 days (Figure 4A). The extended, 7-day assay reflects the fact that HCMV replicates and spreads slowly, with a single cycle of growth extending over 72–96 hours in MRC-5 cells. FLS-359 was well tolerated by confluent MRC-5 cells over 7 days, when assayed by counting of nuclei (Figure 4B) or neutral red uptake (Figure 4C); and it was also tolerated by dividing MRC-5 cells over 6 days (Supplemental Figure 4). FLS-359 reduced the total infected cell area marked by mCherry expression in confluent fibroblasts (Figure 4, D and E). Reduced mCherry expression was also evident when monitored by Western blot assay (Supplemental Figure 5), mimicking expression of the viral immediate-early protein IE1. This assay indicates that FLS-359 inhibits HCMV spread with an IC_50_ of 0.466 ± 0.203 μM (Figure 4F). Control anti-HCMV drugs acting at different steps in the viral replication cycle, ganciclovir and letermovir, exhibited IC_50_s of 1.7 and 0.003 μM, respectively, consistent with literature reports (57, 58). The potencies measured by the spread assay were similar to potencies determined by 50% tissue culture infectious dose (TCID_50_) assay of virus in the medium (Figure 4G).

Although letermovir efficiently reduced extracellular infectivity (Figure 4G), it reduced infected cell area (Figure 4F) to a lesser extent (4-fold) than ganciclovir (33-fold) or FLS-359 (>100-fold). Part of this effect could result from a failure of letermovir to eliminate originally infected cells. However, letermovir blocks at a late point in the viral replication cycle, inhibiting the virus-coded terminase subunit pUL56 (59, 60), which cleaves a unit genome of viral DNA as it enters the capsid. Earlier work has shown that HCMV capsids can spread directly from cell to cell (55), and a portion of the residual spread observed with letermovir might result from movement of partially assembled DNA-capsid complexes.

To test whether inhibition of HCMV is a general feature of SIRT2 inhibitors, we assayed tool compounds with chemical structural diversity: AGK2 (27), AK-7 (61), SirReal2 (49), MIND4 (38), and TM (46). Although they were less potent than FLS-359, all of the SIRT2 inhibitors reduced HCMV spread (Table 2 and Supplemental Figure 6). Since the inhibitors have very different structures, this result argues that they all inhibit HCMV at least in part through targeted modulation of SIRT2.

Having demonstrated the broad-spectrum antiviral attributes of FLS-359, including anti-herpesvirus activity, we focused on the drug’s parameters as an anti-HCMV agent.

### FLS-359 reduces HCMV spread when administered after an infection has been initiated.

**Figure 5:**
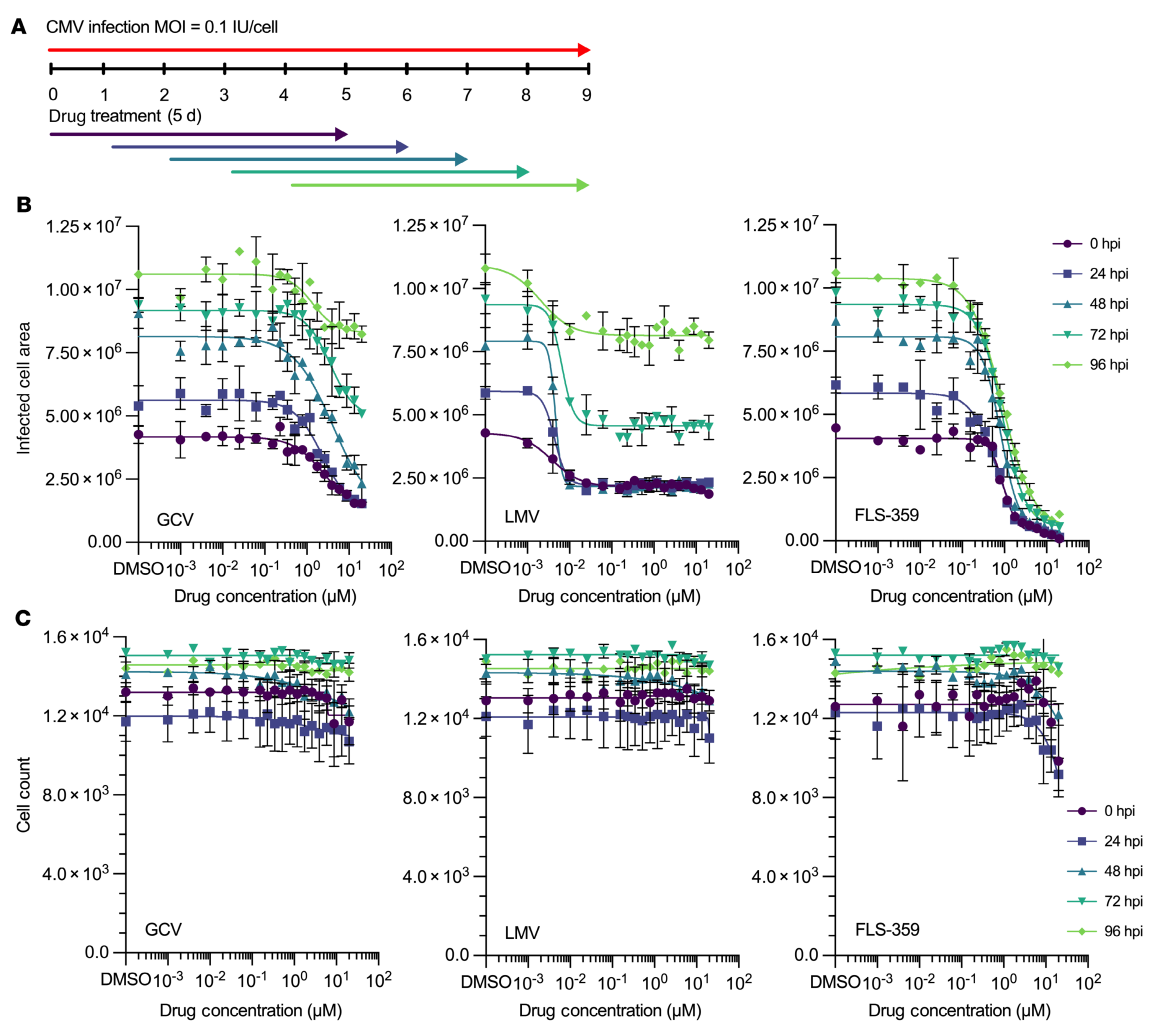
FLS-359 is effective in a delayed treatment protocol. (**A**) Confluent MRC-5 cells were infected with TB40/E-mCherry-UL99eGFP (0.1 IU/cell). FLS-359, ganciclovir (GCV), or letermovir (LMV) was added at 0 hpi, or delayed for 24, 48, 72, or 96 hours. (**B** and **C**) After 5 days of drug treatment, infected cell area was quantified by mCherry fluorescence (**B**) and cell counts by nuclear DAPI stain (**C**). Mean ± SD is shown (*n* = 3).

To model the ability of FLS-359 to control an established infection in comparison with current standards of care, MRC-5 fibroblasts were infected (0.1 IU/cell) and treated with drug either immediately or after a delay of 1–4 days (Figure 5A). Drug treatment was maintained for 5 days, and then the effect of the delay was assayed by monitoring of viral spread. FLS-359, ganciclovir, and letermovir each exhibited antiviral activity when administered after a delay (Figure 5B) without inducing toxicity at effective doses (Figure 5C). Indeed, the antiviral IC_50_s remained essentially unchanged as the addition of drug was delayed over increasing intervals (Supplemental Table 3). At each delayed time of drug addition, a greater maximal fold reduction of infected cell area was achieved by treatment with FLS-359, compared with ganciclovir or letermovir (Supplemental Table 3). Thus, FLS-359 maintains its antiviral potency when administered at 4 days after the initiation of infection, while demonstrating superior control of viral spread compared with standards of care.

### FLS-359 induces an antiviral state that persists after the drug is withdrawn.

**Figure 6:**
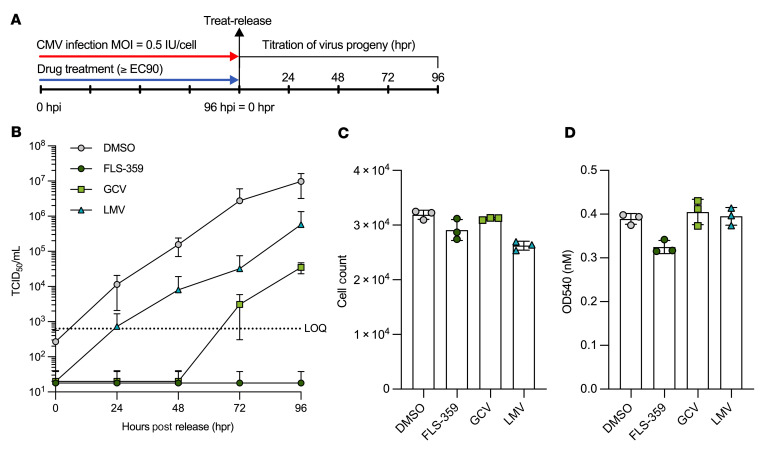
Long-term antiviral activity of FLS-359 following removal of drug. (**A**) Confluent MRC-5 cells were infected with TB40/E-mCherry-UL99eGFP (0.5 IU/cell) in the presence of drugs at their approximate EC_90_s (FLS-359, 5 μM; ganciclovir [GCV], 20 μM; letermovir [LMV], 0.05 μM). Infection proceeded for 96 hours, monolayers were washed 3 times with buffer (PBS), and drug-free growth medium was added. Supernatant was sampled at 24-hour intervals over 4 days after release, and virus was titered by TCID_50_. (**B**) Cell-free virus titers as a function of time after release of the drug-induced block. LOQ, limit of quantification. (**C** and **D**) Cell viability was assessed at 96 hours after release of the drug block by cell count (**C**) or lactate dehydrogenase activity (**D**). Mean ± SD is shown (*n* = 3).

Long-lasting consequences of SIRT2 modulation could result from a long intracellular half-life of the compound or from modifications to the cellular acylome. Accordingly, we performed a treat-release experiment in which MRC-5 cells were infected (0.5 IU/cell) for 96 hours in the presence of drug, drug was removed, and virus growth was monitored by TCID_50_ assay of culture medium for an additional 96 hours (Figure 6A). Cells treated with vehicle (DMSO) lacking drug served as a control, and continued to produce virus throughout the time course (Figure 6B). Release of the letermovir-induced block generated detectable progeny at 24 hours, the first time monitored after release, with a resumption of growth kinetics similar to that in DMSO-treated cultures; release of the ganciclovir block generated detectable progeny at 48 hours; and release from the FLS-359 block did not generate progeny over the full 96-hour period that was monitored. Minimal cellular toxicity was evident in FLS-359–treated cultures at 96 hours after removal of the drug (Figure 6, C and D), arguing against a nonspecific toxic effect.

The long-term efficacy of FLS-359 after washout in the treat-release protocol with only a portion of cells infected suggested that the drug might protect uninfected cells from subsequent infection. This possibility was tested in a treat-release-infect experiment in which MRC-5 cells were treated with drug for 24 hours, drug was removed for 72 hours, and then cells were infected (0.5 IU/cell) and infected cell area was measured at 72 hours post-infection (hpi) (Supplemental Figure 7A). Whereas 24-hour pretreatment with FLS-359 followed by a 72-hour washout inhibited viral spread (IC_50_ = 4.8 μM), ganciclovir had no activity in the same pretreatment regimen (Supplemental Figure 7B). In a control experiment, both drugs were active when readministered at the time of infection (Supplemental Figure 7C). This indicates that FLS-359 alters the susceptibility of cells to infection, but does not speak to the mechanism.

The long-term efficacy of FLS-359 could also result from a prolonged intracellular half-life. Therefore, we monitored its half-life in uninfected MRC-5 cells by treating them with drug for 24 hours, removing medium with drug and washing the cells, and then quantifying drug levels by mass spectrometry (Supplemental Figure 8A). A reduced but significant level of FLS-359 was detected in cells (~5 μM) and supernatant (~0.4 μM) at 72 hours after drug removal, whereas the control drug, letermovir, was effectively removed from supernatant and cells by washing (Supplemental Figure 8, B and C). As a control, FLS-359 was added to medium in cell culture dishes without cells, and it was efficiently removed by washing (Supplemental Figure 8, D and E), ruling out the possibility that the drug was simply sticking to the plastic dishes. FLS-359 induces a relatively long-lasting pharmacodynamic effect in both infected and uninfected cells that inhibits HCMV replication, at least in part due to an extended intracellular half-life.

### FLS-359 inhibits the accumulation of intracellular HCMV RNAs and DNA and reduces the infectivity of virus progeny.

**Figure 7:**
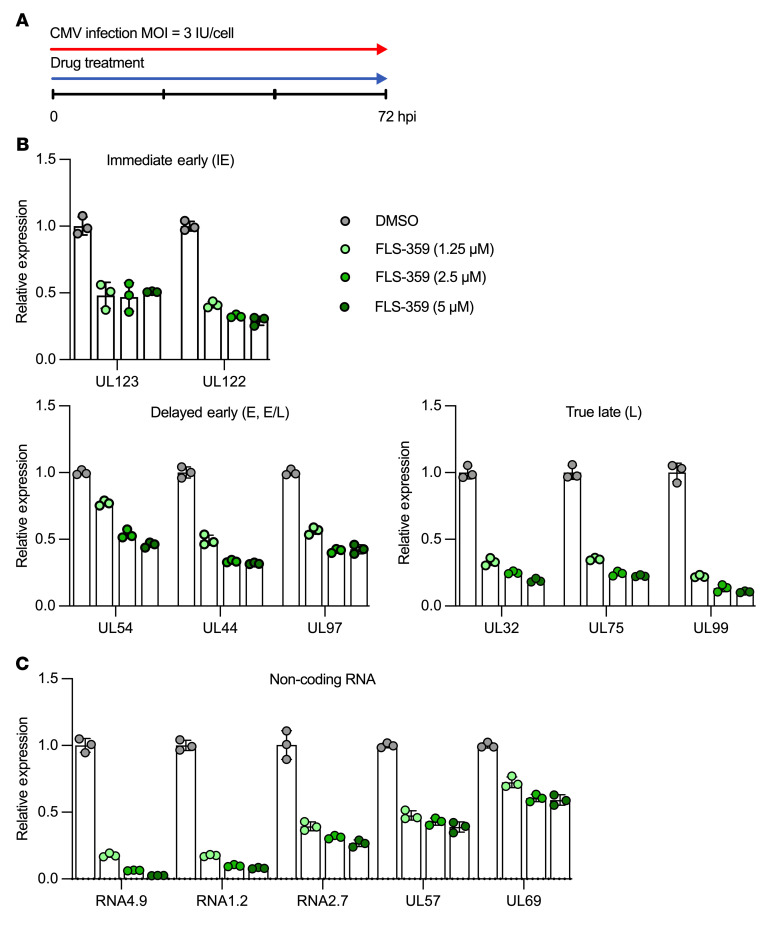
FLS-359 suppresses accumulation of all kinetic classes of viral RNAs. (**A**) MRC-5 cells were mock-infected or infected with TB40/E-mCherry-UL99eGFP (3 IU/cell). FLS-359 was added at indicated doses following adsorption, cell RNA was prepared at 72 hpi, and viral RNAs were quantified relative to cellular GAPDH RNA by qRT-PCR. (**B**) Dot plots showing that FLS-359 reduces accumulation of all tested protein-coding RNAs, including multiple representatives of each kinetic class. (**C**) Dot plots showing that FLS-359 reduces expression of multiple viral noncoding and protein-coding RNAs. Mean ± SD is shown (*n* = 3).

To evaluate the site in the viral replication cycle that is sensitive to SIRT2 inhibition, we monitored the accumulation of representative immediate-early, early, and late HCMV protein-coding RNAs. MRC-5 cells were infected (3 IU/cell), cells were harvested, and RNAs were quantified by quantitative reverse transcriptase PCR (qRT-PCR) assay at 72 hpi (Figure 7A). All of the viral protein-coding RNAs tested were reduced by the SIRT2 inhibitor, and in most cases, the reduction was dose dependent (Figure 7B). The immediate-early UL123 (IE1) and UL122 (IE2) RNAs encode master regulators that modulate expression of the other viral genes (62, 63), so it is possible that an inhibitory event reducing their levels propagates to reduce accumulation of the remaining genes that were assayed.

**Figure 8:**
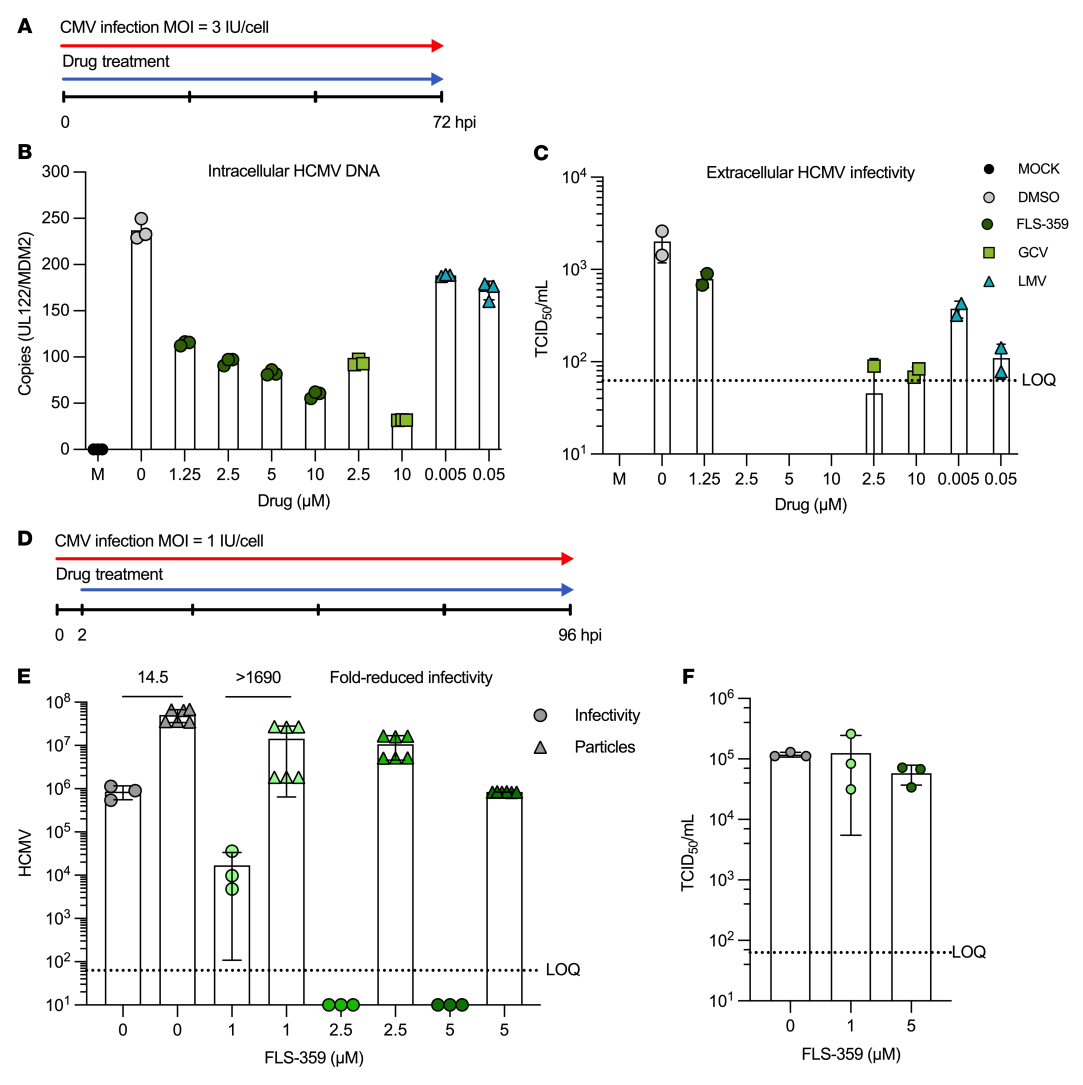
FLS-359 reduces intracellular HCMV DNA accumulation and extracellular virus production. (**A**) MRC-5 cells were mock-infected or infected with TB40/E-mCherry-UL99eGFP (3 IU/cell), treated with the indicated FLS-359, ganciclovir (GCV), and letermovir (LMV) doses, and harvested at 72 hpi. (**B**) Cellular DNA was prepared, and HCMV DNA was quantified by qPCR using UL122-specific probes and normalized to cellular MDM2 DNA (*n* = 3). (**C**) The effect of drugs on virus yield was monitored by TCID_50_ assay (*n* = 2). (**D**) MRC-5 cells were infected with TB40/E-mCherry-UL99eGFP (1 IU/cell) and treated with the indicated drug doses from 2 hpi to 96 hpi. (**E**) DNase I–resistant viral DNA was quantified by qPCR (*n* = 6), and infectious virus was quantified by TCID_50_ assay (*n* = 3). (**F**) TB40/E-mCherry-UL99eGFP virus (10^5^ IU/mL) was incubated with indicated drug doses for 24 hours, and infectious virus was quantified by TCID_50_ assay (*n* = 3). LOQ, limit of quantification. Mean ± SD is shown.

FLS-359 also reduced the level of HCMV-coded long noncoding RNAs, including RNA4.9, which crosses the viral origin of DNA replication (oriLyt), as well as the UL57 and UL69 protein-coding RNAs that flank the oriLyt (Figure 7C). Reduced accumulation of RNA4.9 restricts HCMV DNA accumulation (64). In addition, UL44, UL54, and UL57 — all of which are reduced by drug treatment — encode products that function directly in viral DNA replication (65). UL44 and UL54 encode subunits of the viral DNA polymerase, and UL57 encodes a single-stranded DNA-binding protein. Not surprisingly, then, intracellular viral DNA accumulation was compromised by treatment with FLS-359 for 72 hours (Figure 8A) in a dose-dependent manner (Figure 8B). As expected, ganciclovir, a 2′-deoxyguanosine analog, also inhibited intracellular viral DNA accumulation, when tested using doses equivalent to its IC_50_ and IC_90_. In contrast, letermovir, which acts at a post-replication step, did not have a significant effect on DNA accumulation. FLS-359 reduced the production of infectious virus to below the limit of quantification, whereas ganciclovir and letermovir reduced virus yield to a more limited extent, as expected for the doses tested (Figure 8C). To further evaluate the effect of FLS-359 on virus production, cells were infected, drug treatment was initiated at 2 hpi and maintained until 96 hpi, and then the infectivity of progeny virus particles was evaluated (Figure 8D). As seen for the 72-hour treatment (Figure 8C), virus infectivity was dramatically reduced by the 96-hour drug treatment (Figure 8E). At 1.0 μM FLS-359, the particle/infectious unit ratio was reduced by a factor of 14.5, while 2.5 or 5.0 μM drug reduced infectivity more than 1,690-fold. In a control experiment, incubation of a virus stock with FLS-359 (1 or 5 μM) for 96 hours at 37°C had no significant effect on infectivity (Figure 8F), ruling out the possibility that the drug inactivates virions. Thus, FLS-359 reduces the accumulation of intracellular viral nucleic acids, the production of extracellular virus particles, and the infectivity of the virus particles that are generated.

### FLS-359 inhibits HCMV infection in 2 humanized mouse models.

FLS-359 pharmacokinetics was assessed in female BALB/c mice (Supplemental Table 4). After a single 50 mg/kg oral (p.o.) dose, the drug exhibited an approximately 6-hour plasma half-life, achieving maximal plasma concentrations (C_max_) of 89 μM, substantially exceeding the in vitro IC_50_s. The relatively long half-life and high C_max_ resulted in good exposure, with an AUC of 713 μM•h/mL. FLS-359 was also administered to NOD/Shi-scid/IL-2Rγ^null^ (NOG) mice at 50 mg/kg p.o. twice per day (b.i.d.) for 14 days. No weight loss and no adverse clinical signs were observed, indicating that FLS-359 is well tolerated at this dose and schedule.

**Figure 9:**
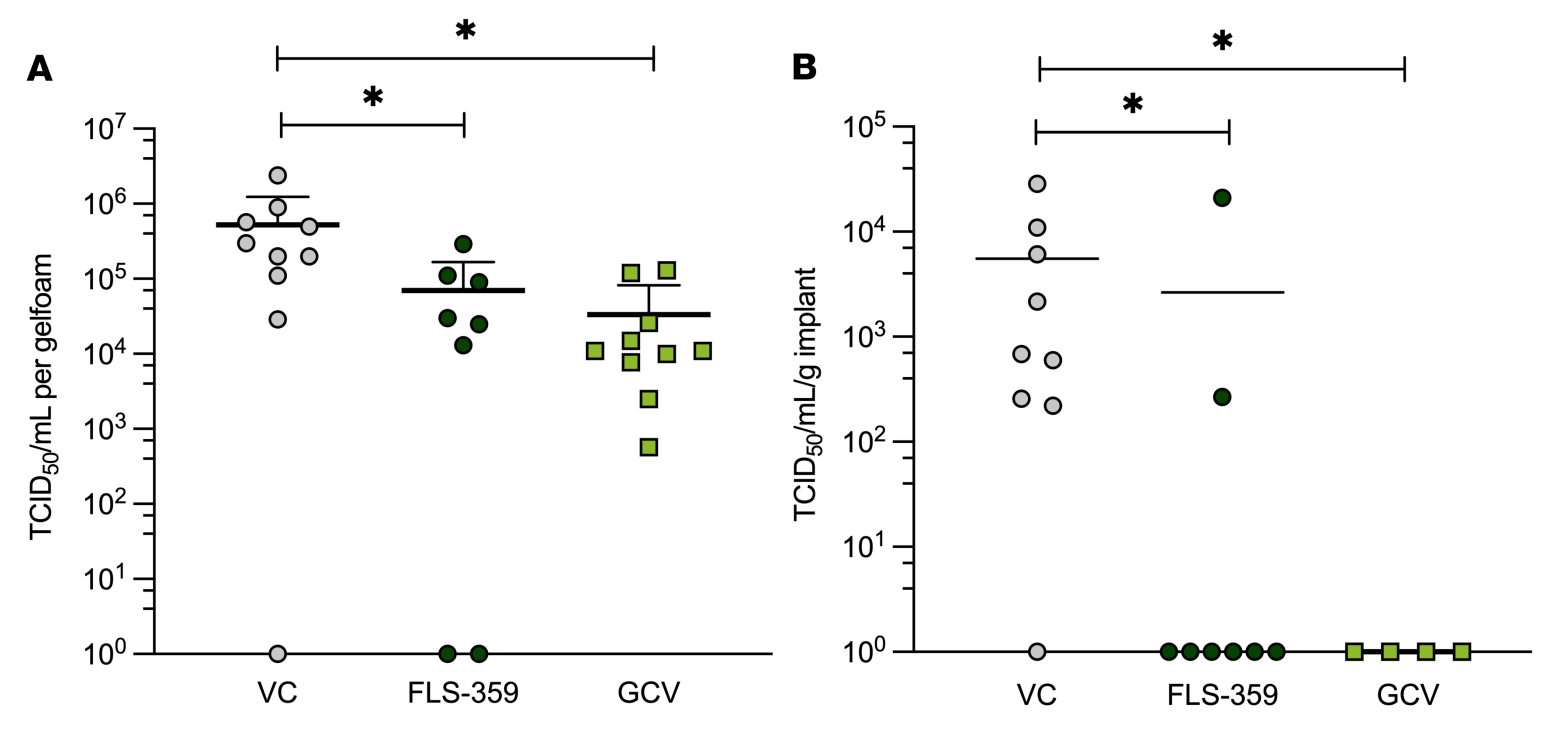
In vivo efficacy of FLS-359. (**A**) Gelfoam/human fibroblast model. MRC-5 cells were infected with TB40/E-mCherry-UL99eGFP (0.05 IU/cell), incubated for 24 hours, harvested, counted, and seeded (1 × 10^6^ cells) into gelfoam plugs. After 3 days of incubation, plugs were implanted subcutaneously on the flanks of CIEA NOG mice. Beginning at 24 hours after implantation, mice were treated with vehicle control (VC; 0.5% methylcellulose + 0.5% Tween-80, p.o., b.i.d.), FLS-359 (50 mg/kg in VC, p.o., b.i.d.), or valganciclovir (GCV; 100 mg/kg in VC, p.o., b.i.d.). After 11 days of treatment, gelfoam plugs were harvested, and virus was quantified by TCID_50_ assay. *P < 0.03. (**B**) Human lung-only mouse model. Beginning at 2 hours before infection with TB40/E (4.25 × 10^5^ IU) via direct injection into lung implants, lung-only mice were treated with vehicle control (VC; 0.5% methylcellulose + 0.5% Tween-80 in sterile distilled water, p.o., b.i.d.), FLS-359 (50 mg/kg in VC, p.o., b.i.d.), or ganciclovir (GCV; 100 mg/kg in VC, i.p., daily). After 17 days of treatment, implants were harvested and processed for TCID_50_ determination. *P < 0.04. Variance was calculated by 1-sided Kruskal-Wallis test followed by Dunn’s multiple-comparison test.

The inhibitory activity of FLS-359 was tested in 2 humanized models of HCMV infection. The first (58, 66) used TB40/E virus–infected MRC-5 fibroblasts (0.05 IU/cell), seeded into a collagen matrix (gelfoam) and then implanted subcutaneously (1 × 10^6^ infected cells) into immunodeficient mice. FLS-359 (50 mg/kg, p.o., b.i.d.), valganciclovir (50 mg/kg, p.o., daily), or diluent was administered beginning immediately after implantation. Implants were recovered on day 11 after infection, and TCID_50_ assays revealed that both drugs significantly reduced virus production (Figure 9A). We also tested the efficacy of FLS-359 in humanized lung-only mice, generated by subcutaneous implantation of human lung tissue into immune-deficient mice (67). In this model, the human lung tissue expands to form a highly vascularized palpable implant that contains human fibroblast, epithelial, endothelial, and mesenchymal cells, which form lung-like structures that support HCMV replication and in vivo efficacy testing of antiviral agents (67, 68). Administration of FLS-359 (50 mg/kg, p.o., b.i.d.), ganciclovir (100 mg/kg, i.p., daily), or diluent was initiated 2 hours before infection of human lung implants by direct inoculation of TB40/E virus (4.25 × 10^5^ IU/implant). Drug treatments were continued until the lung implants were removed on day 17 after infection, and virus was quantified by TCID_50_ assay. Both drugs again significantly reduced the production of infectious HCMV progeny (Figure 9B), confirming in vivo activity of the SIRT2-targeted drug.

## Discussion

FLS-359 bound SIRT2 between its Zn^++^-binding domain and the catalytic Rossmann fold domain (Figure 2), in the EC pocket where other small-molecule ligands have been localized (49). A thermal shift assay (Figure 1B) and inhibition of deacetylase activity (Figure 1, C and D) confirmed drug binding. FLS-359 decreases SIRT2 deacetylase activity, but SIRT2 remains partially active when fully occupied by drug, as seen with other tool compounds, e.g., AGK2 (27) and MIND4 (38), possibly because FLS-359 sits in the EC pocket, but the acetyl-peptide substrate can still bind and undergo catalysis, albeit at a reduced rate. This explanation is consistent with the small decrease in SIRT2 activity at 10× *K_m_* concentration of acetyl-substrate peptide (Figure 1E), and argues that the drug is an allosteric modulator. The movement in the FLS-359/SIRT2 crystal structure (Supplemental Figure 2) when compared with apo structure (ref. 50; PDB ID 3ZGO) is consistent with this allosteric binding mechanism.

FLS-359 binds and modulates SIRT2 in vitro (Figure 1) and within cells (Supplemental Figure 1), but does FLS-359 antiviral activity result from SIRT2 modulation? Although we have documented robust anti-HCMV activity for FLS-359 (Figure 4), it has been reported that knockdown of SIRT2 increases the yield of HCMV by a factor of about 4 at 96 hpi (24). This apparent contradiction likely results from the different functional consequences of a knockout/knockdown that ablates all SIRT2 activities, as compared with SIRT2-modulating drugs that exhibit substrate selectivity (40–43). SIRT2 removes a variety of long-chain acyl groups in addition to acetyl groups in biochemical assays (19, 20), and FLS-359 blocks SIRT2 deacetylation but not demyristoylation (Figure 1F and Supplemental Table 1). Modeling predicts that SIRT2 can accommodate both an acetyl group and FLS-359, whereas the binding of a myristoyl group excludes FLS-359 (Supplemental Figure 3). As a consequence, FLS-359 can modulate SIRT2 deacetylation, but cannot inhibit demyristoylation activity; and it is possible that additional acylations escape inhibition by the drug. Substrate-selective drugs have been described for a variety of enzymes (42), and knockdown/knockout experiments cannot reliably predict the physiological consequences of their activities. One approach that addresses this experimental conundrum is to test the activity of multiple, different drugs that modulate a specific enzymatic activity (42), as we have done for the anti-HCMV activity of SIRT2 inhibitors (Figure 4F, Table 2, and Supplemental Figure 6). Six structurally distinct SIRT2-targeting compounds reduced the yield of HCMV in spread assays, arguing that SIRT2 modulation is a key element of the antiviral activity.

It is conceivable that these compounds have off-target activities that impact viral growth, perhaps via effects on additional members of the SIRT family. However, this possibility seems unlikely, because the thioacyl lysine TM has been tested in vitro against all seven SIRTs and is highly selective for SIRT2 (46). Further, FLS-359 (Figure 1C), AGK2 (69), MIND4 (38), and SirReal2 (49) are highly selective for SIRT2 versus their most closely related family members, SIRT1 and SIRT3 (70).

It is not clear why the anti-HCMV activity of FLS-359 (0.5 μM; Figure 4F) appears to be more potent than its in vitro activity on purified SIRT2 (3.3 μM; Figure 1C). Intracellular SIRT2 is produced from 3 splice variants (71), modified by phosphorylations and acetylations (72–74), and associates with numerous other cellular proteins (75). These variations, modifications, and associations might make SIRT2 more or less susceptible to inhibition by the drug. Alternatively, the compound might accumulate preferentially in a cellular compartment where a key target protein resides. It is also possible that a secondary target of the drug contributes to its antiviral activity or a more active metabolite is generated within cells. Finally, it is conceivable that inhibition of SIRT2 modulates multiple pathways required for efficient viral replication, amplifying the antiviral effect of the drug.

Our work highlights the broad-spectrum antiviral activity of FLS-359 (Table 1), which inhibits the replication of both RNA and DNA viruses. The IC_50_s for inhibition of the different viruses tested range from 0.3 μM for SARS-CoV-2 to 6.7 μM for respiratory syncytial virus. This range of sensitivities might result from testing in different cell types with different antiviral readouts. It is also possible that different SIRT2-controlled posttranslational modifications impact different viruses to a greater or lesser extent and the antiviral mechanisms vary across a range of pathogens. This data set clearly illustrates the broad-spectrum activity of this class of inhibitors, a potentially invaluable feature of antivirals in immunosuppressed populations and as new pathogens emerge and evolve in the human population.

How does SIRT2 inhibition antagonize HCMV replication? As noted above, HCMV infection induces profound alterations to the cytoplasmic and nuclear acetylomes, including changes to viral and cellular transcriptional regulatory proteins (15). Some of these changes likely support viral growth and spread, by facilitating viral replication processes or by reducing the cellular protective response. As a consequence, drugs that modulate the infected-cell acetylome, and more broadly the infected-cell acylome, have potential to create an environment that antagonizes viral replication.

Although SIRT2 is predominantly cytoplasmic, it shuttles between the nucleus and cytoplasm, acting in both compartments (76). FLS-359 modestly reduced the accumulation of all viral RNAs tested, including the UL122 and UL123 RNAs (Figure 7B), arguing that it modulates viral transcription or RNA stability. Expression of the UL122 and UL123 RNAs is controlled by the major immediate-early promoter (MIEP), which is the primary HCMV promoter/enhancer to become active following infection. Numerous cellular factors bind at the MIEP (63), and the activity of any of these factors could potentially be modulated by SIRT2 inhibitors. SIRT2 also acts on transcription by modulating the acetylation state of histones H3 and H4 (77, 78). Although SIRT2 inhibition could lead to hyperacetylation of HCMV chromatin, a state that generally favors transcriptional activation, an indirect consequence, such as enhanced expression of a cell-coded repressor, could inhibit viral transcription. Reduced accumulation of the major immediate-early proteins could then reduce the expression of all downstream viral RNAs — similar to what was observed (Figure 7B). Changes in acetylation status of virus-coded factors, such as pUL26, where an acetylation mimic (K203Q) inhibited virus production (15), could also play a role.

FLS-359 reduced intracellular viral DNA accumulation (Figure 8B), a likely consequence of reduced UL44, UL54, UL57, and RNA4.9 RNA expression (Figure 7, B and C). pUL44 and pUL54 are subunits of the viral DNA polymerase, pUL57 is a single-stranded DNA-binding protein required for viral DNA replication, and RNA4.9 is a noncoding RNA that forms an R-loop at the viral origin of DNA replication and is required for efficient viral DNA accumulation (64). However, the relatively modest approximately 3-fold reduction in intracellular viral DNA levels (Figure 8B) does not account for the more than 1,000-fold reduction in infectious virus (Figure 8C) caused by treatment with FLS-359 over the course of 72 hours. A second experiment recorded an approximately 70-fold reduction in extracellular virus particles, and viral infectivity was again reduced by more than 1,600-fold (Figure 8E). Thus, the number of virus particles and their infectivity are both impacted by the drug. Reduced levels of viral proteins could interfere with efficient DNA packaging into capsids, and export of mature enveloped virions. FLS-359 could also interfere with the production of infectious virions by perturbing the microtubule network. Consistent with the role of SIRT2 in α-tubulin deacetylation (44), FLS-359 can induce hyperacetylation of α-tubulin (Supplemental Figure 1A). Altered acetylation of α-tubulin K40 has potential to modulate microtubule activity (79), which in turn is critical for structure (80) and function (81) of the HCMV assembly zone, the viral organelle in which capsids are assembled into virions (82). Further, very-long-chain fatty acids are required for infectivity of virus particles (83), and SIRT2 inhibition impacts lipid synthesis (84), so it is possible that an effect of FLS-359 on fatty acid synthesis reduces the infectivity of HCMV progeny. Additional viral processes, such as nuclear egress of capsids, which is controlled by lamin B1 acetylation (15), might also be impacted by SIRT2 inhibition. Finally, in stressed tumor cells SIRT2 inhibition has been shown to activate p53 (85), induce degradation of overexpressed c-Myc (45, 46), and block full activation of the PI3K/Akt pathway (86) — any of which could potentially reduce the production of infectious viral progeny. Although further studies are needed to more fully appreciate the mode of FLS-359 antiviral action, it appears likely that its anti-HCMV mechanism is multifactorial, and the multiple components likely speak to its broad-spectrum antiviral activity and strongly predict that drug-resistant mutants will not evolve.

Will SIRT2 inhibitors prove to be well tolerated in humans? Mice appeared healthy and alert and did not lose weight during our anti-HCMV studies; SIRT2-knockout (30) and SIRT2/3-knockout (31) mice are healthy; and EX-527, which is selective for SIRT1 but also has anti-SIRT2 activity (87), is well tolerated in humans (88). Further, the acute nature of many viral infections will require short-term treatments, mitigating possible long-term toxicity.

The broad-spectrum antiviral activity of SIRT2 inhibitors can potentially find utility in multiple clinical applications. Treatment of viral disease in transplant patients is a prime example. These immunosuppressed patients have heightened susceptibility to environmental pathogens as well as adventitious agents traveling with donor tissues or resident in the recipient, including herpesviruses, polyomaviruses, respiratory viruses, hepadnaviruses, and emerging viruses (8, 9, 89, 90). We have already determined that several of these viral agents are inhibited by FLS-359, including HCMV (Figure 4), which continues to threaten transplant recipients in spite of effective direct-acting therapies. EBV (Figure 3), HBV, respiratory viruses that include influenza and now SARS-CoV-2, and other newly emerging agents such as Zika virus are also inhibited by FLS-359 (Table 1). A host-targeted, broad-spectrum drug should improve outcomes, especially for transplant patients undergoing antiviral prophylaxis.

In sum, FLS-359 is a representative of a new family of SIRT2 modulators. Its broad-spectrum and multifaceted antiviral activity illustrates its potential as a host-targeted antiviral with utility in treatment of numerous viral diseases and sets the stage for further understanding of how epigenetic mechanisms impact the growth and spread of multiple viral pathogens.

## Methods

### SIRT2 thermal stability assay.

Equal volumes of 2× FLS-359 and 2× human SIRT2^2–352^ (GenScript) in binding buffer (20 mM PIPES, pH 7.4, 100 mM NaCl, 0.005% Tween, 10 μM 1-anilino-8-naphthalenesulfonate) were added to PCR plates (Hard-Shell 384, black well, Bio-Rad), overlaid with silicon oil, and centrifuged (600*g*, 20 seconds). Thermal melt data were collected using custom instrumentation (Fluorescence Innovations), with either 405 nm or 532 nm laser excitation and fluorescence lifetime emission to measure total well fluorescence and fluorescence lifetime as a function of temperature. Data were treated as previously described (37) to determine the midpoint transition temperature, *T_m_*.

### SIRT deacylase assay.

Human SIRT deacylase activity was measured in assay buffer (50 mM Tris, pH 8.0, 137 mM NaCl, 2.7 mM KCl, 1 mM MgCl_2_, 1 mg/mL BSA) containing purified, recombinant SIRT2^2–389^, SIRT1^1–747^, or SIRT3^1–400^ protein (GenScript); acetylated (Ac-H_3_K_9_WW: QTARK^Ac^STGGKAPRWW-NH_2_) or myristoylated (Myr-H_3_K_9_WW: QTARK^Myr^STGGKAPRWW-NH_2_) peptide (GenScript); NAD^+^; and inhibitors. Reactions were initiated by addition of SIRT protein, and aliquots were quenched with 1% formic acid after 10 minutes at 37°C. Reaction products (deacetylated peptide ions or Ac-ADP-ribose) were detected using RapidFire High Throughput Mass Spectrometry (PureHoney Technologies). Deacetylated peptide substrate (GenScript) and Ac-ADP-ribose (Toronto Research Chemicals) were used as controls in detection reactions.

### X-ray structure determination.

The study was performed at Crelux GmbH. SIRT2^56–356^ was used for crystallization; a hexahistidine tag used for purification was removed before crystallization. Crystals of SIRT2 in complex with FLS-359 were obtained using hanging-drop vapor diffusion setups. SIRT2 (21.9 mg/mL; 50 mM HEPES-NaOH, 150 mM NaCl, pH 8.0) was preincubated with 3.6 mM (5.7-fold molar excess) of FLS-359 for 1 hour. A 1 μL aliquot of the protein solution was then mixed with 2 μL of reservoir solution (0.1 M HEPES-NaOH, pH 6.6, 0.3 M Li_2_SO_4_, 21 % [wt/vol] PEG 3350) and streak-seeded before being equilibrated at 20°C over 0.2 mL of reservoir solution. Well-diffracting crystals grew as thick aggregates of thin plates and were mounted within 19 days.

For data collection, crystals were cryoprotected by the addition of ethylene glycol to a concentration of 20% (vol/vol) to the crystallization drop before mounting. Single thin plates were isolated for data collection. A complete 1.8 Å data set of a SIRT2/FLS-359 single crystal was collected at PETRA III (Hamburg, Germany, beamline P11) (Supplemental Table 2), and the data were integrated, analyzed, and scaled by XDS (91) (within the autoPROC pipeline [ref. 92], Pointless (93), and Aimless (94), respectively.

For structure determination and refinement, molecular replacement was done using a Crelux reference structure of SIRT2 as a starting model. Several rounds of alternating manual rebuilding and refinement with REFMAC5 (95) resulted in the final model (Supplemental Table 2). Atomic displacement factors were modeled with a single isotropic B-factor per atom, except for selected cysteine sulfur atoms for which residual electron density after isotropic refinement indicated an anisotropic behavior, as well as for the Zn^++^ atom in chain A. Non-crystallographic symmetry restraints were used throughout the refinement cycles.

Computational superpositioning predictions used the Glide module of Schrödinger, release 2022-4. The following SIRT2 structures were tested for predicted interactions with FLS-359: PDB ID 4RMI and 4RMG (49), 3ZGO (50), 4R8M (96), and 4X3P (97).

Coordinates and structure factors of the SIRT2/FLS-359 complex were deposited in the Protein Data Bank (PDB ID 7T1D).

### Cells, viruses, and reagents.

Human embryonic lung fibroblasts (MRC-5; ATCC CCL-171) were maintained in DMEM with 10% FBS. The type I B lymphoma cell line Akata (52) and MDA-MB-231 breast adenocarcinoma cells (ATCC HTB-26) were cultured in RPMI 1640 medium with 10% FBS. HepG2 hepatocellular carcinoma cells (ATCC HB-8065) were propagated in DMEM with 10% FBS. TB40/E-mCherry-UL99eGFP virus was described previously (73), and was titered by TCID_50_ assay on MRC-5 cells (98). FLS-359 was synthesized as described in US Patent Application US20210139475A1. ^1^H NMR was consistent with the structure, and purity was determined to be greater than 97% by reversed-phase HPLC. Ganciclovir and AGK2 (MilliporeSigma), letermovir (MedChem Express), SirReal2 and MIND4 (Chembridge/Hit2Lead), AK-7 (Cayman Chemical), and TM (Abmole) were stored at –20°C as 10 mM stocks in DMSO.

### Assays for FLS-359 antiviral activity.

SARS-CoV-2 (strain USA-WA1/2020) was assayed on Calu3 cells by qRT-PCR quantitation of extracellular viral genomes using remdesivir as an antiviral control at the United States Army Medical Research Institute of Infectious Diseases (USAMRIID). Zika virus (strain DAK41525) was assayed on human foreskin fibroblasts by immunofluorescence assay detecting a viral antigen using amodiaquine as an antiviral control at USAMRIID. HCMV (strain TB40/E) was assayed on MRC-5 fibroblasts by spread assay using ganciclovir and letermovir as antiviral controls (Figure 4). Influenza A (strain A/California/07/2009) was assayed on differentiated normal human bronchial epithelial cells by yield reduction assay using ribavirin as an antiviral control by a Division of Microbiology and Infectious Diseases contractor. Betacoronavirus 1 (strain OC43) was assayed on MRC-5 fibroblasts in a cytopathic effect (CPE) inhibition assay at Evrys Bio. Junin virus (strain Candid 1) was assayed on MRC-5 fibroblasts by immunofluorescence assay detecting a viral antigen using RIID E-1 as an antiviral control at USAMRIID. Hepatitis B virus (genotype D, subtype ayw) was assayed on primary human hepatocytes by monitoring of viral relaxed circular DNA using tenofovir as an antiviral control at ImQuest Biosciences. Epstein-Barr virus (EBV; strain Akata) was assayed on Akata BL cells activated by treatment with anti-IgG, and then viral gp350 expression was monitored using phosphonoacetic acid (PAA) as an antiviral control (Figure 3). Respiratory syncytial virus (strain Long) was assayed on MRC-5 fibroblasts by immunofluorescence assay detecting multiple viral antigens using ribavirin as an antiviral control at RetroVirox. Assays were performed in triplicate to determine IC_50_s.

### Assay for HCMV spread.

Confluent MRC-5 cultures were infected with TB40/E-mCherry-UL99eGFP (0.01 IU/cell). Drugs were added after a 1-hour adsorption period using a Tecan D300e dispenser, and the DMSO concentration was normalized to 0.5% across wells. At 7 days post-infection (dpi), fluorescent images were captured using a BioTek Cytation 3 Multi-Mode Reader and analyzed using Agilent BioTek Gen5 software to calculate infected cell area. Uninfected, drug-treated cultures were fixed with 4% paraformaldehyde, stained with DAPI, imaged, and analyzed to determine nuclei counts.

### Protein, RNA, and DNA analysis.

Proteins were analyzed by Western blot as previously described (99) using anti-mCherry (1:1,000; EPR20579, Abcam), anti-IE1 (1:100; clone 1B12; ref. 100), anti–acetyl-α-tubulin K40 (1:10,000; T7451, MilliporeSigma), anti–c-Myc (1:2,000; Y69, Abcam), anti–β-actin (1:10,000; A5441, MilliporeSigma), and anti–α-tubulin (1:10,000; DMA1, MilliporeSigma) primary antibodies, plus IRDye 680RD anti-mouse or IRDye 800CW anti-rabbit (1:20,000; LI-COR) secondary antibodies. (See full, uncut gels in the supplemental material.) RNA and DNA were analyzed by qPCR assay as previously described (99) using primers listed in Supplemental Table 5.

### Analysis of mouse pharmacokinetic parameters and tolerability.

FLS-359 was formulated as a suspension at 5 mg/mL in 0.5% methylcellulose (cP 25) plus 0.5% Tween-80 (MilliporeSigma) in sterile water and vortexed and/or sonicated immediately before p.o. administration. The pharmacokinetics study used fed female BALB/c mice (22 g, *n* = 3 per time point). Blood samples (~30 μL via saphenous vein puncture) were taken at 0.25, 0.5, 1, 2, 4, 8, and 24 hours. A standard curve was prepared in control plasma using FLS-359 (0.01–10 μg/mL) in terfenadine solvent (50 ng/mL in methanol/acetonitrile 1:1 vol/vol). The lower limit of quantification was 10 ng/mL. An aliquot of 10 μL plasma sample was mixed with 10 μL terfenadine solvent plus 2 μL methanol. An additional 200 μL of terfenadine solvent was added, and the resulting mixture was vortexed for 1 minute and centrifuged at 1,500*g* for 15 minutes. The supernatant was diluted 10× with methanol/water (1:1 vol/vol with 0.1% formic acid), and 2 μL aliquots were analyzed using a QTRAP 4000 LC-MS/MS System (Sciex) with a C18 column (Kinetex). Pharmacokinetic parameters were determined with the noncompartmental analysis tool in WinNonlin (Certara).

To assess the tolerability of FLS-359, female NOD/Shi-scid/IL-2Rγ^null^ (NOG) mice were treated with 50 mg/kg p.o., b.i.d., for 14 days. Mice had free access to food and water and were evaluated for morbidity and mortality twice daily. Body weights and food consumption were recorded once per day before the morning dosing. Detailed clinical observations were made 1–2 hours after the morning compound administration and once 5–6 hours after the morning observation.

### Mouse gelfoam-fibroblast model for anti-HCMV activity.

For the mouse gelfoam model (58, 66), MRC-5 cells were infected with HCMV TB40/E-mCherry-UL99eGFP (0.05 IU/cell). On the same day, sterile gelfoam (SURGIFOAM Absorbable Gelatin Sponge, USP) was aseptically cut into 1.2 cm × 0.5 cm × 0.7 cm pieces and transferred into a sterile dish containing DMEM. Infected cultures and gelfoam pieces were incubated at 37°C for 24 hours. Then, infected cells were harvested and counted, and 1 × 10^6^ cells in medium (30 μL) were slowly added to the gelfoam pieces in a 24-well non–tissue culture–treated plate. Seeded gelfoams were incubated for 3 hours at 37°C, and then 1 mL of medium was added to each gelfoam and incubated at 37°C for 3 days. On the day of gelfoam implant, 18-week-old male and female NOD.Cg-*Prkdc^scid^Il2rg^tm1Sug^/JicTac* mice (CIEA NOG, Taconic Biosciences) were anesthetized using isoflurane (Patterson Veterinary), the dorsal area above the hip region was shaved and sterilized with Betadine (Purdue Products) and 70% ethanol, the gelfoam was inserted beneath the skin, and incisions were closed. FLS-359 (50 mg/kg, p.o., b.i.d.), valganciclovir (50 mg/kg, p.o., daily), or diluent (0.5% methylcellulose plus 0.5% Tween-80) was administered, beginning immediately after implantation. On day 11, mice were euthanized, the gelfoams were harvested, homogenized, and clarified by centrifugation at 17,500*g* for 5 minutes at 4°C, and virus in the supernatant was quantified by TCID_50_.

### Human lung-only mouse model for anti-HCMV activity.

Lung-only mice (LoM) were generated as previously described (67, 68). In brief, LoM were constructed by implanting of 2 pieces of human lung tissue (Advanced Bioscience Resources) subcutaneously into the back of male and female NOD.Cg-*Prkdc^scid^Il2rg^tm1Wjl^/SzJ* mice (NSG, The Jackson Laboratory). Expansion of the implants was monitored by palpation. Anesthetized mice were exposed to HCMV by direct injection of HCMV TB40/E (4.25 × 10^5^ IU) into the implants in a total volume of 100 μL. Mice received vehicle control (0.5% methylcellulose, 0.5% Tween-80; p.o., b.i.d.), FLS-359 (50 mg/kg in vehicle; p.o., b.i.d.), or ganciclovir (100 mg/kg; i.p., daily) beginning 2 hours before infection. Human lung implants were harvested at 17 dpi and flash-frozen. Subsequently, implants were thawed and homogenized, and virus load was measured by TCID_50_ assay.

### Statistics.

Quantitative results are shown as mean ± SD of independent experiments as noted in the figure legends. Statistical significance was evaluated using GraphPad Prism 8 (GraphPad Software). Efficacy readouts in animal studies were compared between treatment groups by Kruskal-Wallis test, followed by 1-sided Dunn’s multiple-comparison test. A *P* value of 0.005 was used to determine statistical significance.

### Study approval.

Mouse studies were carried out in compliance with the NIH *Guide for the Care and Use of Laboratory Animals* (National Academies Press, 2011) according to protocols approved by Institutional Animal Care and Use Committees of Bioduro Beijing Co. (protocol BD-201709136, mouse pharmacokinetics), the Trudeau Institute (protocol 19-002, HCMV gelfoam), and the University of North Carolina at Chapel Hill (protocol 20-235, HCMV LoM).

## Author contributions

KLR, SR, MJT, JLK, LT, APB, CD, WWR, AVW, JVG, MAL, TS, EAM, and LWC designed research studies. KLR, SR, MJT, LT, APB, AVW, and CD conducted experiments and acquired data. KLR, SR, MJT, JLK, LT, APB, CD, WWR, AVW, JVG, MAL, TS, EAM, and LWC analyzed data. KLR, SR, MJT, JLK, LT, AVW, APB, WWR, AW, JVG, MAL, TS, JRT, EAM, and LWC contributed to writing the manuscript and/or preparing figures.

## Supplementary Material

Supplemental data

## Figures and Tables

**Table 1:**
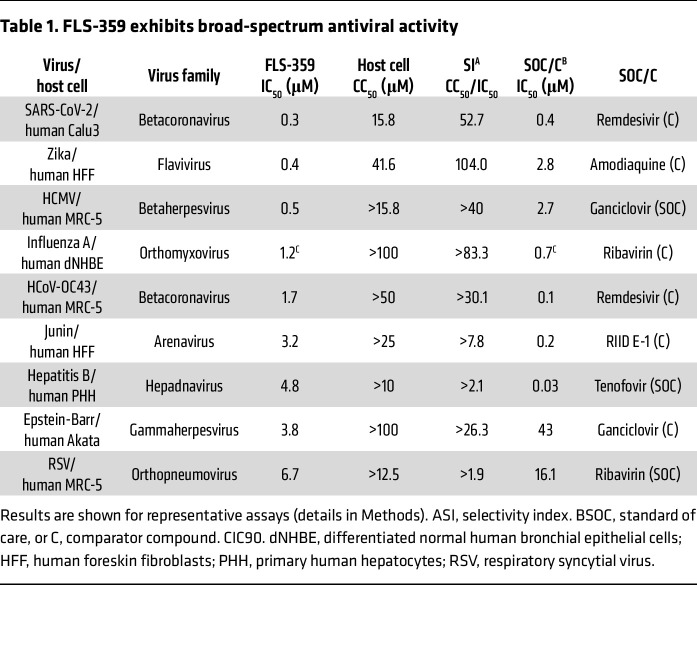
FLS-359 exhibits broad-spectrum antiviral activity

**Table 2:**
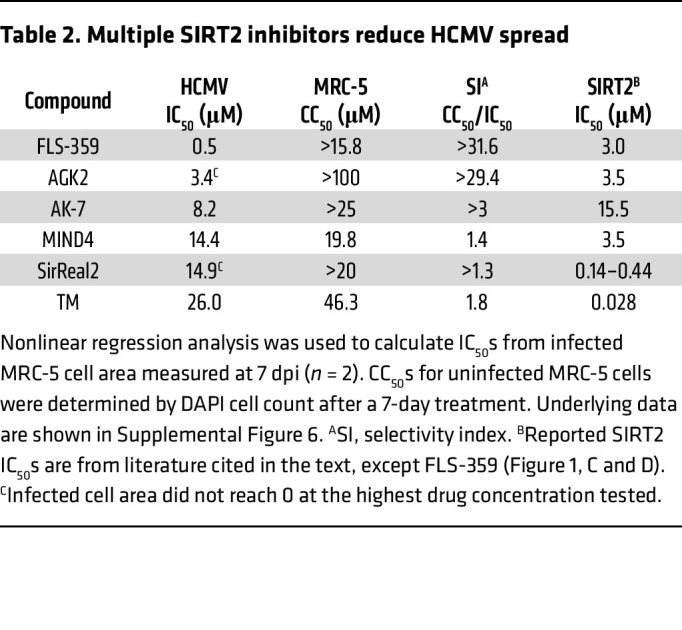
Multiple SIRT2 inhibitors reduce HCMV spread

## Version 1 06/15/2023

Electronic publication
