# Supplementary material for: An allosteric inhibitor of sirtuin 2 deacetylase activity exhibits broad-spectrum antiviral activity
Source: J Clin Invest. 2023 Jun 15;133(12):e158978. doi: 10.1172/JCI158978 (PMC10266789; doi:10.1172/JCI158978)

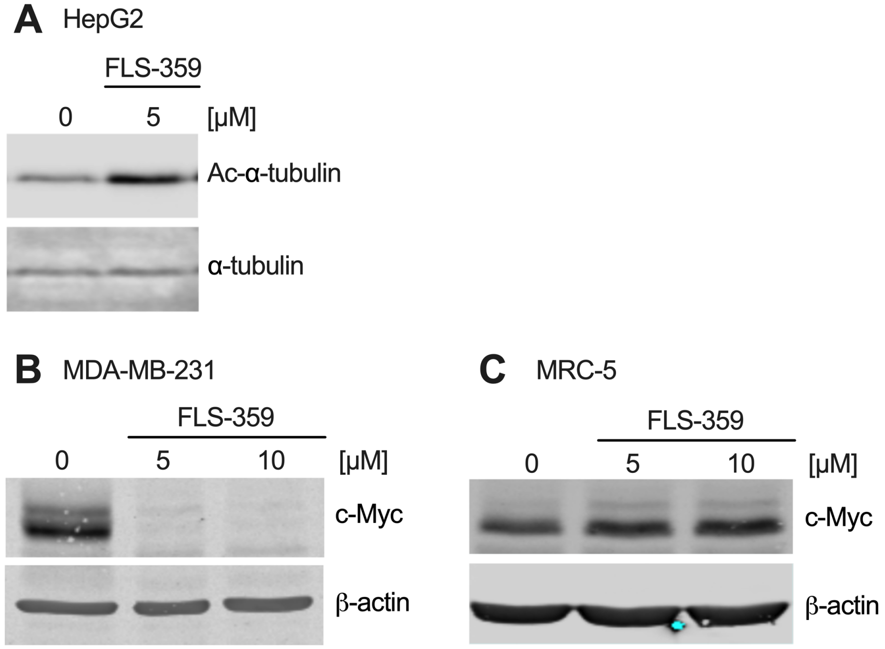

Supplemental Figure 1. FLS-359 is active in cultured cells. (**A**) HepG2 cells were treated with FLS-359 plus 1 µM trichostatin A (TSA) for 24 h and levels of acetylated a-tubulin (Ac-a-tubulin) were determined by western blot. Total a-tubulin was monitored as a control. (**B**) MDA-MB-231 cells or (**C**) MRC-5 fibroblasts were treated with drug for 72h and c-Myc was monitored by western blot with b-actin as a control. Western blots are representative of three independent experiments.

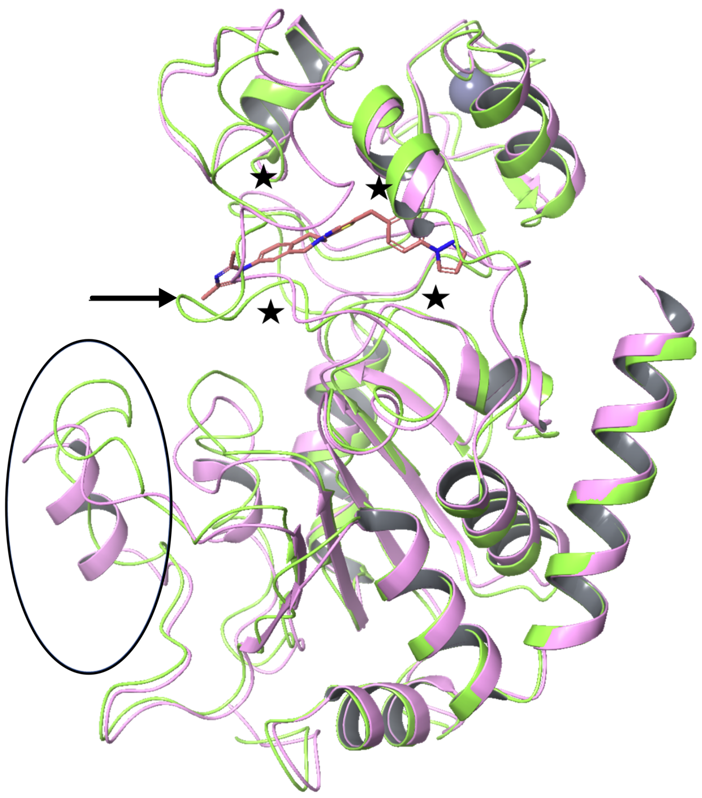

Supplemental Figure 2. Shifts of SIRT2 apo structure upon FLS-359 binding. The oval highlights residues that are in an alpha helix confirmation in the apo structure (purple ribbons, PDB ID 3ZGO) but shift to an unstructured loop with FLS-359 binding (green ribbons). The arrow identifies a shift in a loop over the drug binding site, between the apo and FLS-359-bound SIRT2 structures. Stars mark the domain (center of the clamshell) that opens when FLS-359 is bound.

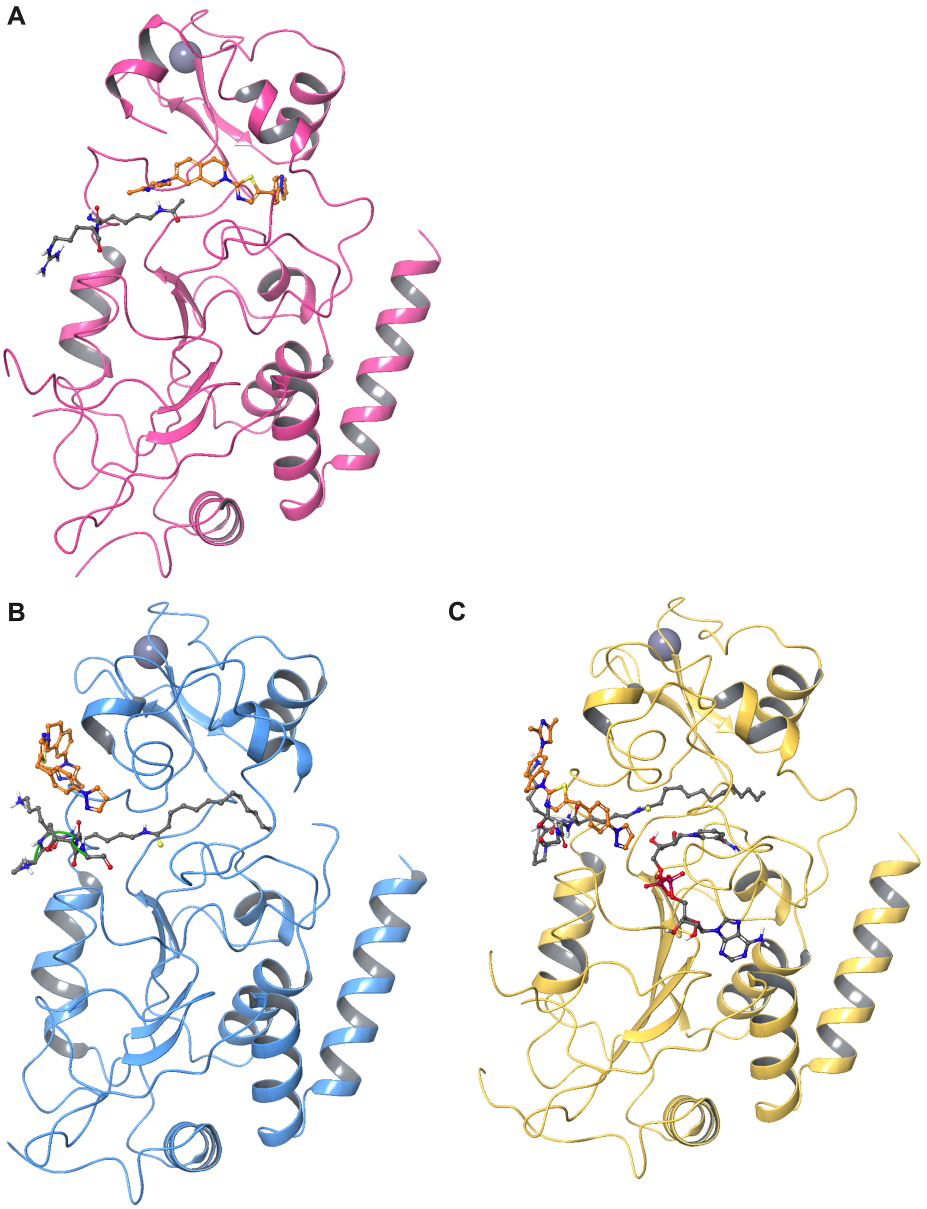

Supplemental Figure 3. Docking analysis predicts that a myristoylated peptide competes with FLS-359 for binding to SIRT2. The interaction of FLS-359 with known SIRT2-acyl peptide structures was analyzed by flexible protein docking using Glide, a module of the Schrödinger software.
(**A**) Acetylated peptide (TGGK^{Ac}APR; gray carbon atoms) bound to SIRT2 (PDB ID 4RMI) does not block FLS-359 (orange carbon atoms) binding in the EC site. (**B**) Thiomyristoylated peptide (PKK^{TMy}TG; gray carbon atoms) bound to SIRT2 (PDB ID 4R8M) displaces FLS-359 to the protein surface.
(**C**) Thiomyristoylated peptide plus NAD (blue carbon atoms) bound to SIRT2 (PDB ID 4X3P) displaces FLS-359.

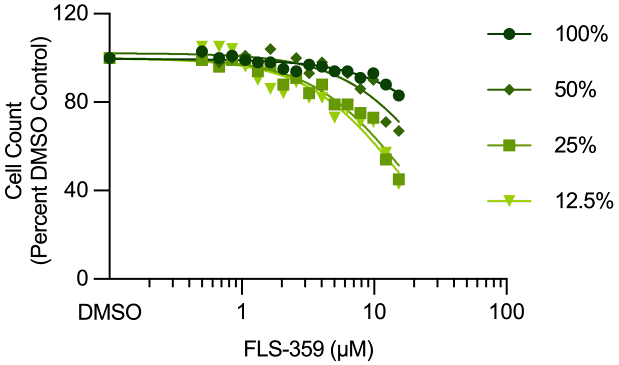

Supplemental Figure 4. FLS-359 does not significantly inhibit the growth of MRC-5 fibroblasts at the IC_{90} for inhibition of HCMV. Uninfected MRC-5 cells were plated to achieve the indicated initial levels of confluence, treated with FLS-359, and counted after 6 days, or approximately two cell doublings. FLS-359 IC_{50} (0.5 mM) and IC_{90} (2.06 mM) in Figure 4 were determined on 100% confluent cells.

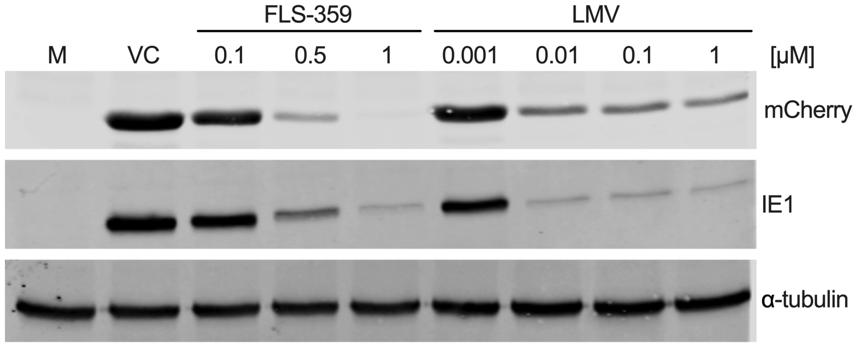

Supplemental Figure 5. FLS-359 and letermovir reduce the accumulation of an HCMV-coded immediate-early protein. MRC-5 cells were mock-infected or infected with TB40/E mCherry UL99-eGFP (0.01 IU/cell) and treated with FLS-359 or letermovir (LMV). Cell lysates were harvested at 7 dpi, and mCherry, IE1, and a-tubulin were detected by western blot; M, mock-infected; VC, vehicle control. This image is representative of two independent experiments.

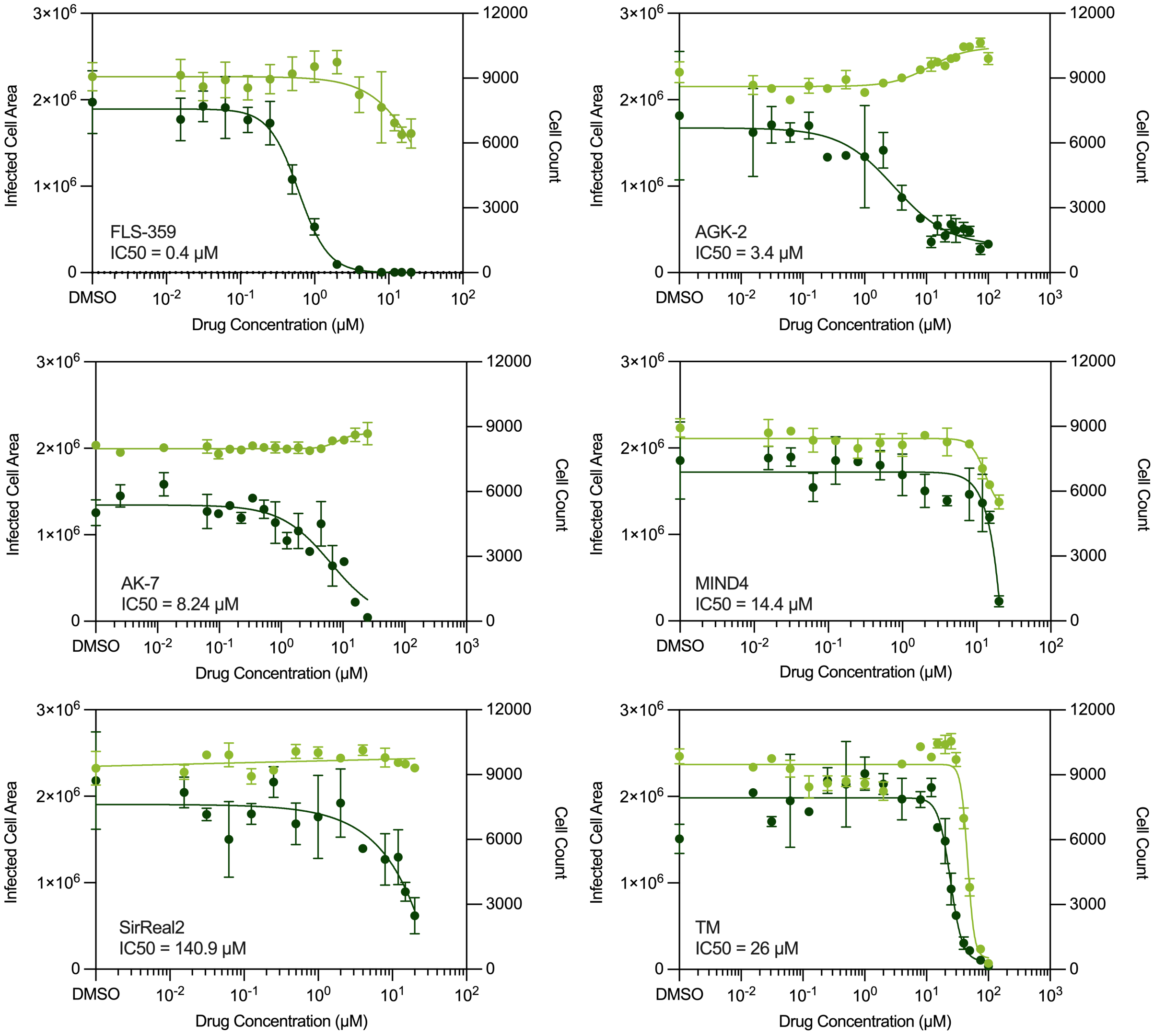

Supplemental Figure 6. Multiple SIRT2 inhibitors block HCMV growth. Confluent MRC-5 cells were infected with TB40/E-mCherry-UL99eGFP (0.01 IU/cell). Drug or vehicle (DMSO) was added after inoculation and the fluorescent area marking infected cells was quantified at 7 dpi (dark green circles). Cell viability was monitored by staining drug-treated (7 days), uninfected MRC-5 cells with DAPI and quantifying nuclei (light green circles). Mean +/- SD is shown (n=3).

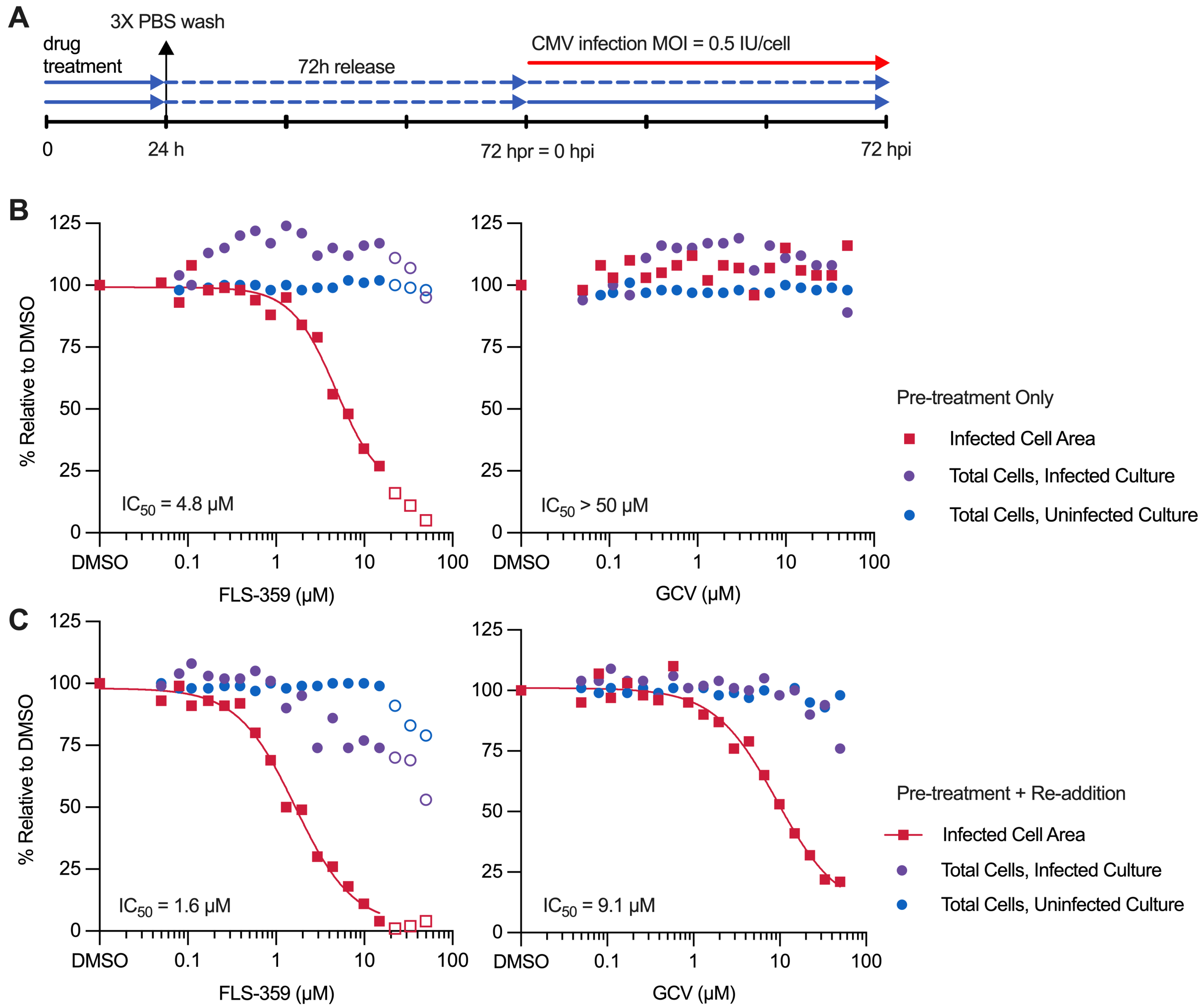

Supplemental Figure 7. Pre-treatment of uninfected cells with FLS-359 followed by a drug-free period protects against subsequent HCMV infection. (A) MRC-5 cells were pre-treated with FLS-359 or GCV for 24h followed by a 72h drug-free release period. Then they were infected with TB40/E-mCherry-UL99eGFP (0.5 IU/cell) in the absence of drug or with drug re-addition. Uninfected cells, drug-treated in the same manner, served as a control. **(B-C)** Infected cell area was quantified by mCherry fluorescence and cell counts by nuclear DAPI stain at 72 hpi. Results are representative of three independent experiments.

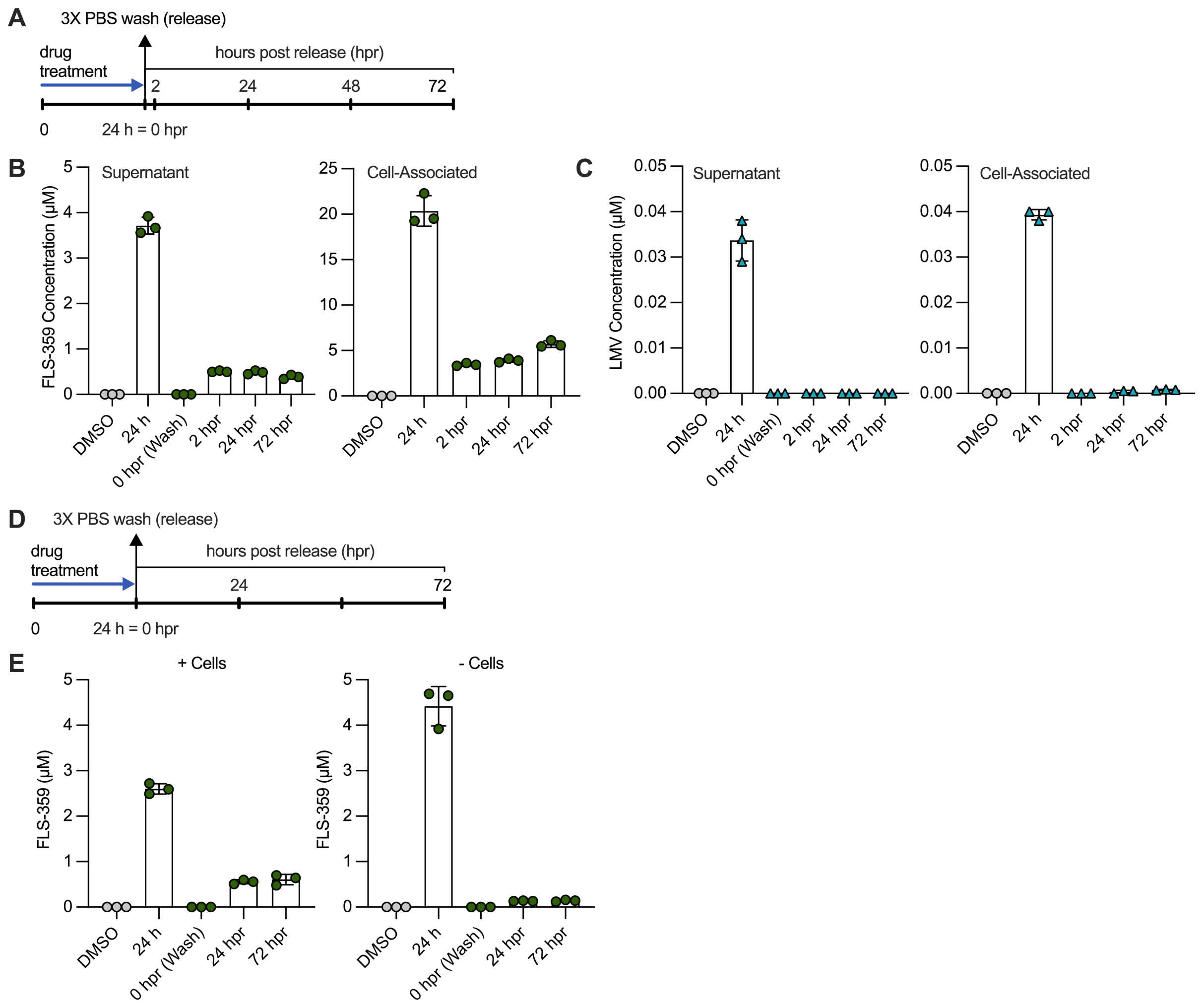

Supplemental Figure 8. FLS-359 exhibits a long half life within MRC-5 cells. (**A**) Uninfected cells were treated with drug (FLS-359 = 5 μM; LMV = 0.05 μM). After 24h, supernatant was collected, monolayers were washed 3x to remove drug and cells were replenished with drug-free media. Supernatants and cells were then assayed for residual drug levels at 2, 24 or 72h post-release. (**B-C**) Supernatant and cell-associated drug concentrations were determined by mass spectrometry. (**D**) Medium containing 5 μM FLS-359 was added to plastic tissue culture dishes with or without a confluent monolayer of MRC-5 cells. Sampling of the supernatant was conducted after a 24h incubation at 37°C, followed by 3X washes with PBS, collection of the final wash, and replenishment of drug-free medium to the dishes. Supernatant samples were then collected at 24 and 72h post drug-removal. (**E**) FLS-359 drug concentrations as determined by mass spectrometry. Mean +/- SD is shown (n=3).

Supplemental Table 1. Deacylation activities of FLS-359 and anti-SIRT2 tool compounds.

Drug	Deacetylase			Demyristoylase
	IC_{50} (µM)	pIC_{50} ± SE	NTV (%)	IC_{50} (µM)
FLS-359	3.0	5.5 ± 0.1	6.5 ± 4.0	>100
SirReal2	0.2	6.7 ± 0.1	1.5 ± 1.3	>100
AGK2	0.5	6.3 ± 0.3	39 ± 11	>100

The negative log of the IC_{50} (pIC_{50}) and percent SIRT2 activity with saturating compound (Net Terminal Value, NTV), are reported as averages for triplicate measurements with standard errors. Enzymatic reactions were initiated by the addition of SIRT protein, quenched after 10 min at 37°C and deacylated product peptide H3K9WW was detected by mass spectrometry. FLS-359 data is from the experiment shown in Figure 1F.

Supplemental Table 2. FLS-359-SIRT2 Data Collection Statistics.

	Parameter	Value
Data Collection Statistics	Crystal identifier	xctc3-fls-03-08
	Inhibitor	FLS-359
	Space Group	$P2_1$
	Unit cell parameters (Å, °)	a=36.8, b=56.0, c=139.6, α=90.0, β=94.5, γ=90.0
	Resolution (Å)	139.15-1.75 (1.78-1.75)
	# Unique reflections	56312 (3005)
	I/σ/I	9.5 (1.9)
	Completeness (%)	98.6 (98.9)
	Multiplicity	3.5 (3.4)
	R_{meas}	0.09 (0.73)
	R_{pim}	0.05 (0.39)
	CC(1/2)	0.996 (0.664)
Refinement Statistics	Resolution (Å)	139.15-1.75 (1.78-1.75)
	R_{work}	0.159 (0.236)
	R_{free}	0.210 (0.284)
	Completeness (%)	98.3 (98.6)
	r.m.s.d. bonds (Å)	0.009
	r.m.s.d. angles (°)	1.56

Numbers in parentheses refer to the highest resolution bin.

Supplemental Table 3. FLS-359 is effective in a delayed-treatment protocol.

Time of Drug Addition	IC_{50} (μM)			Maximum Fold-Reduction		
(hpi)	GCV	LMV	FLS-359	GCV	LMV	FLS-359
0	2.5	0.004	0.9	3.2	2.0	11.6
24	2.3	0.004	0.6	4.5	2.7	33.4
48	4.1	0.005	0.8	7.5	3.7	21.2
72	3.6	0.007	0.9	1.9	2.0	17.6
96	1.4	0.002	0.9	1.3	1.3	21.8

Confluent MRC-5 cells were infected with TB40/E-mCherry-UL99eGFP (0.1 IU/cell). FLS-359, ganciclovir (GCV) or letermovir (LMV) was added at 0 hpi, or delayed for 24, 48, 72, or 96 h. After 5 days of drug treatment, infected cell area was quantified by mCherry fluorescence and cell counts by nuclear DAPI stain, as shown in Figure 5. Results report IC_{50} and maximum fold-reduction in infected cell area.

Supplemental Table 4. FLS-359 pharmacokinetic profile.

Dose (mg/kg)	50
C_{max} (μM)	89.3 ± 11
T_{max} (h)	2.0 ± 0
$T_{1/2}$ (h)	5.8 ± 1.4
$AUC_{0-\infty}$ (μM·h/mL)	713.0 ± 33
$AUC_{0-\infty}$ /1 mg dose	14.3 ± 1.0

To evaluate FLS-359 pharmacokinetics, a single dose of FLS-359 was administered (p.o.) to female Balb/c mice as a suspension in 0.5% methylcellulose/0.5% Tween80/water. Plasma samples from groups of three animals were assayed over the course of 24h. Mean +/- SD is presented.

Supplemental Table 5. Primers used in quantitative PCR assays.

Transcript	Forward (5'-3')	Reverse (5'-3')
SetDB1	GACTACAATACCGGGACAGTAGC	CCCAGCATCACCTGAATCAAT
BZLF1	AGCCTGCTCCTGAGAATGCT	CCACTGCTGCTGCTGTTTGA
BMRF1	CGTGCCAATCTTGAGGTTTT	CGGAGGCGTGGTTAAATAAA
BLLF1	CCCGCTGGACTTTTACGA	GCATGGAGAGGTTTGAGA
UL123	GCCTTCCCTAAGACCACCAAT	ATTTTCTGGGCATAAGCCATAATC
UL122	ATGGTTTTGCAGGCTTTGATG	ACCTGCCCTTCACGATTCC
UL99	GCTGCGGCTCTGCGGTAG	GCGAAACGTCGAGCGCAC
UL97	CGGCGTCACCACTTTGACC	CGTCACGCATCACGTCACTT
UL75	TCTCCGTCGTATGCACCAGC	GATCGCCGACTTTGCCCTAC
UL69	GCAGTGCCACGAGTGTCA	GGTGAGATCCAATAGCGACTGCT
UL57	TGAACGCAGAAACGCAGGAG	GAAATCCGCCTCCACCGTGA
UL54	CCCTCGGCTTCTCACAACAAT	GGCGAGTTAGTCTTGGCCAT
UL44	TACAACAGCGTGTCGTGCTCCG	CATGCGTATCAACGTGCAGCTG
UL32	GGTTTCTGGCTCGTGGATGTCG	CACACAACACCGTCGTCCGATTAC
RNA 4.9	GTAAGACGGGCAAATACGGT	AGAGAACGATGGAGGACGAC
RNA 2.7	TCCATGTTTCCATCCTTTCA	AATCAGCGTTGCAGTAGTCG
RNA 1.2	TGACAACGCCTTGTATAGCC	AGACTGTCGTGGTCGATGAA
GAPDH	ACCCACTCCTCCACCTTTGAC	CTGTTGCTGTAGCCAAATTCGT
MDM2	CCCCTTCCATCACATTGCA	AGTTTGGCTTTCTCAGAGATTTCC

Full unedited gels for Supplemental Figure 1

A

Ac-α-tubulin Western Blot Gel (HepG2 cells)

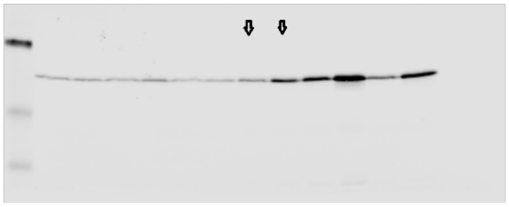

Total α-tubulin Western Blot Gel (HepG2 cells)

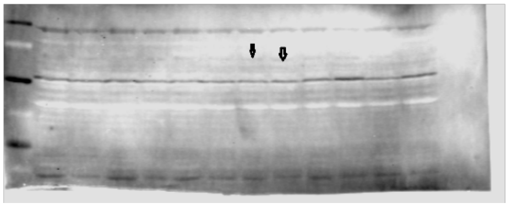

B

cMYC and β-actin Western Blot Gel (MDA-MB-231 cells)

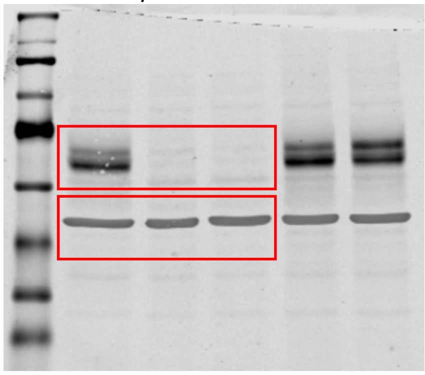

C

cMYC Western Blot Gel (MRC5 cells)

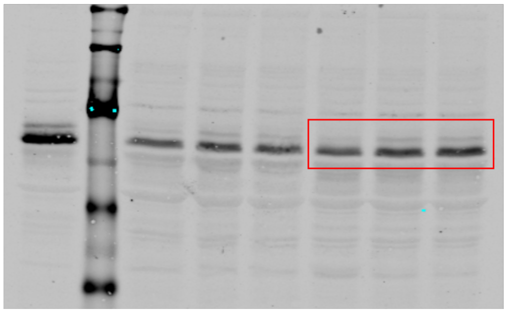

β-actin Western Blot Gel (MRC5 cells)

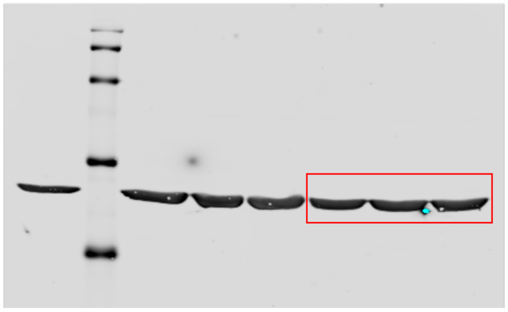

Full unedited gels for Supplemental Figure 5

mCherry Western Blot Gel

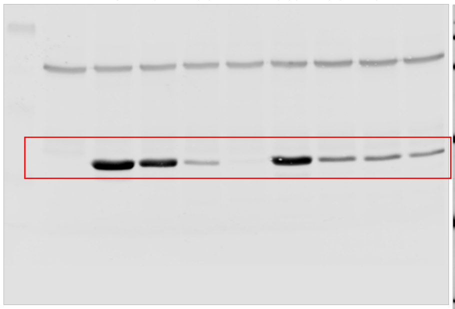

IE1 Western Blot Gel

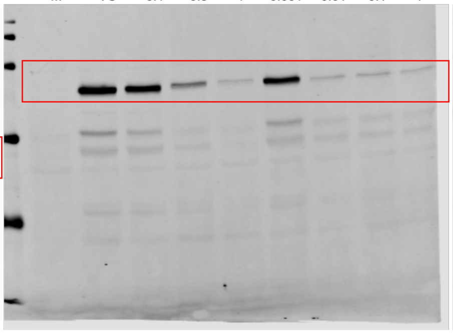

α-tubulin Western Blot Gel

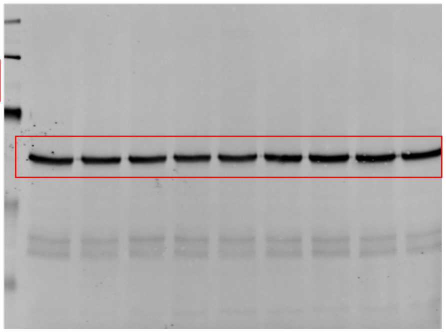

Supplement: Supplemental data [file jci-133-158978-s120.pdf]
